# Historical Maps from Modern Images: Using Remote Sensing to Model and Map Century-Long Vegetation Change in a Fire-Prone Region

**DOI:** 10.1371/journal.pone.0150808

**Published:** 2016-03-30

**Authors:** Kate E. Callister, Peter A. Griffioen, Sarah C. Avitabile, Angie Haslem, Luke T. Kelly, Sally A. Kenny, Dale G. Nimmo, Lisa M. Farnsworth, Rick S. Taylor, Simon J. Watson, Andrew F. Bennett, Michael F. Clarke

**Affiliations:** 1 Department of Ecology, Environment and Evolution, La Trobe University, Bundoora, Victoria, Australia; 2 Arthur Rylah Institute for Environmental Research, Department of Environment, Land, Water and Planning, Heidelberg, Victoria, Australia; 3 School of Life and Environmental Sciences, Deakin University, Burwood, Victoria, Australia; University of Maryland at College Park, UNITED STATES

## Abstract

Understanding the age structure of vegetation is important for effective land management, especially in fire-prone landscapes where the effects of fire can persist for decades and centuries. In many parts of the world, such information is limited due to an inability to map disturbance histories before the availability of satellite images (~1972). Here, we describe a method for creating a spatial model of the age structure of canopy species that established pre-1972. We built predictive neural network models based on remotely sensed data and ecological field survey data. These models determined the relationship between sites of known fire age and remotely sensed data. The predictive model was applied across a 104,000 km^2^ study region in semi-arid Australia to create a spatial model of vegetation age structure, which is primarily the result of stand-replacing fires which occurred before 1972. An assessment of the predictive capacity of the model using independent validation data showed a significant correlation (r_s_ = 0.64) between predicted and known age at test sites. Application of the model provides valuable insights into the distribution of vegetation age-classes and fire history in the study region. This is a relatively straightforward method which uses widely available data sources that can be applied in other regions to predict age-class distribution beyond the limits imposed by satellite imagery.

## Introduction

Remotely sensed data increasingly are being used as a data source to map the results of disturbance, as portrayed in fire history [1,2,3] and stand age maps [4–6], for example. Studies of forest stand-age based on remotely sensed data have predominately been undertaken in temperate *Pinus* forests of the United States of America and Canada [6–9], with some notable exceptions (e.g. Europe [4,10] and South Africa [5]). Near-infra-red reflectance has been shown to decrease with increasing forest age in studies on coniferous forests, due to changes in chlorophyll content, and increasing canopy gaps due to stem mortality [9,11]. These studies have not been replicated in a range of environments, and it is not clear whether a similar relationship between stand age and reflectance would occur in other vegetation types, such as arid and semi-arid vegetation where bare ground is a feature of all age-classes.

Remotely sensed data are also increasingly being used to map fire histories e.g. [1,2,3]. Fire history maps assist land managers to understand the fire history of a region, particularly when produced over broad spatial scales and long time-frames. They provide critical information for land managers and are routinely used for fire planning [12,13], analysing trends in fire characteristics [14], providing historical context for assessing the nature of recent fires [15,16], and identifying and managing important fire age-classes for species conservation [17–19]. Consequently, fire history maps are being developed for ecological management around the world, including in Africa [20], Australia [21,22], North America [13,16], and the Mediterranean [14,23].

Common approaches to producing fire history maps from remotely sensed imagery include identifying recent fires from a single image by manual or automated techniques [21,24], or by detecting differences between two or more years of remotely sensed data to locate fires in the intervening period [1,24,25]. Recent fire scars are usually distinctive on satellite images, particularly when canopy species have been affected. This reduces the need for ground surveys (except for assessment of accuracy), enabling a cost-effective survey. The main limitation of these approaches is the temporal extent of remotely sensed data. The Landsat program commenced in 1972 and provided the first, widely available, global satellite images. However, for many fire-prone regions, large areas of land have not burned since 1972, and thus have little or no fire history information. Because the life-span of many plant species is far greater than 40 years, a true understanding of the ecological response of these vegetation communities to fire requires fire history models that extend over much greater time spans.

Many other types of data have been employed to map fire history over time spans that exceed the availability of satellite imagery (e.g. 100 years or more). Where aerial photography is available, it is a useful data source to extend knowledge of fire history [26,27]. However, aerial photography is not widely available and canopy species may have commenced growing prior to the earliest aerial photography. Data on fires that occurred prior to satellite imagery may also be collated from existing maps, hand-drawn fire boundaries [28]from old survey plans and surveyors’ notes, fire scars on trees [29,30], tree ring analysis [31] and stem diameters. Limitations of these data sources, such as availability over time, spatial coverage, inconsistencies in the exclusion of unburnt islands within the fire boundary and cost, restrict their utility in developing detailed fire history maps over large temporal and spatial scales.

Given these limitations, a number of studies have investigated the use of remotely sensed data for mapping fire regimes over larger temporal scales [13,32–34], although few studies have created historical fire boundaries or time-since-fire maps. A global scale model was developed by Mouillot and Field [34] to reconstruct fire history for the 20^th^ century. It used available fire history, vegetation data and extrapolations from these data, combined with Along Track Scanning Radiometer (ATSR) satellite sensor data. However, this broad scale approach was not designed for regional land management. Hessl et al. [33] successfully used interpolation techniques to map paleo fire boundaries, applied within a relatively small study area with a maximum interpolation area of 750 m from sites of known fire age. The challenge remains to build accurate maps of vegetation age structure in fire-prone environments at a spatial and temporal scale relevant to land management.

Here, we present a method for spatially explicit modelling to predict vegetation age-classes over a large spatial area at a fine scale (25 x 25 m) relevant to land management. We use artificial neural network models (ANN) and remotely sensed data, the latter being widely available, making this technique suitable for determining the age-class distribution of many ecosystems where stand-replacing fires or other broad scale disturbances occur. Neural network models are frequently used for classification of remotely sensed data [35,36]. Jensen et al. [9], for example, found neural networks were suited to modelling stand age from remotely sensed data because of their flexibility in analysing non-linear and non-normally distributed data.

Our study region is the semi-arid Murray Mallee region of south-eastern Australia, characterised by multi-stemmed ‘mallee’ eucalypts. ‘Mallee’ vegetation displays characteristics which make it well suited to this type of analysis. First, there is a close relationship between stand age and fire history because during wildfire the stems of mallee eucalypts typically are killed. Following fire, regeneration occurs from the lignotuber (mallee root), enabling rapid and consistent regrowth of above-ground stems [37]. Second, fire is the major cause of widespread and extensive stand replacement in mallee vegetation, although small areas of stem death may occur through drought, frost [38] wind storm, disease and insect attack. In the post-fire vegetation succession, there are predictable changes in the structural characteristics of the vegetation, including the density of mallee stems, the cover of the hummock grass *Triodia*, and the cover of understory vegetation [39]. These changes occur at broad spatial scales, and are detectable by satellite imagery.

Fires in the Murray Mallee region have been systematically mapped from 1972 onwards [40]. However, areas may remain unburnt for centuries [37], and so fire history maps of the last 40 years do not represent the true distribution of fire age-classes across the region. Using site-specific data, Haslem et al. [39] found that habitat and fuel attributes measured in mallee vegetation remained dynamic beyond 35 years post-fire, and most attributes showed nonlinear patterns of change post-fire. Hence, grouping sites of unknown time-since-fire into a single “old” age class (i.e. the most recent fire was before satellite imagery) does not give a complete picture of the way habitat or fuel changes. Similarly, models of animal occurrence based on a ~100 year post-fire chronosequence showed that some species of reptiles [17], mammals [41] and birds [42] have century-long successional dynamics, extending well beyond the 40 year limit imposed by satellite imagery.

Given the long time-frames over which fire-prone ecosystems may operate, there is a critical need to develop a spatially explicit view of vegetation age beyond the available satellite imagery. Therefore, the aims of this research were to:

1. develop and test a technique to accurately map mallee age-classes in relation to fire prior to 1972,
2. generate a spatial map of age-classes older than 1972 to show the full range of age classes present in the region, and
3. describe the modelling process to allow further investigation and application in other ecosystems.

We successfully developed an artificial neural network model of the relationship between remotely sensed attributes and the fire age of sites. This model was used to produce a spatially explicit map showing the predicted age of mallee vegetation, from 35 to 120 years, across the Murray Mallee region. The approach is described in sufficient detail to allow replication in other ecosystems.

## Methods

Permission was granted by the following agencies and organisations to conduct field surveys: Parks Victoria Department of Environment, Land, Water and Planning (Victoria), NSW National Parks and Wildlife Service Department of Environment and Climate Change (NSW) Department for Environment and Heritage (SA)

This study was conducted as part of a larger project, the Mallee Fire and Biodiversity Project, which investigated the effects of fire on flora and fauna within semi-arid mallee vegetation in south eastern Australia [42]. The study region encompasses 104,000 km^2^ of the Murray Mallee region and spans parts of three Australian states: Victoria, New South Wales and South Australia. Mallee describes vegetation with a canopy layer dominated by multi-stemmed ‘mallee’ *Eucalyptus* species. The understorey varies, with three broad vegetation classes described and mapped as part of the project: Triodia Mallee, Chenopod Mallee and Heathy Mallee [43].The study area has a semi-arid climate with average annual rainfall of between 246–335 mm [44].

Fire is the major stand-replacing disturbance in the mallee region; however, other potential disturbances include grazing, vegetation clearing, wind storm and frost. Of these, windstorm and frost are the only common stand replacing disturbances. Vegetation clearance in this region is usually permanent, because clearing was usually followed by “grubbing” or burning of the lignotuber (mallee root) for three or more consecutive years to prevent rapid regeneration [45]. Grazing of mallee eucalypt coppice after fire is minimal because the nitrogen level and digestibility are low and goats do not find mallee suckers palatable [46].

**Fig 1:**
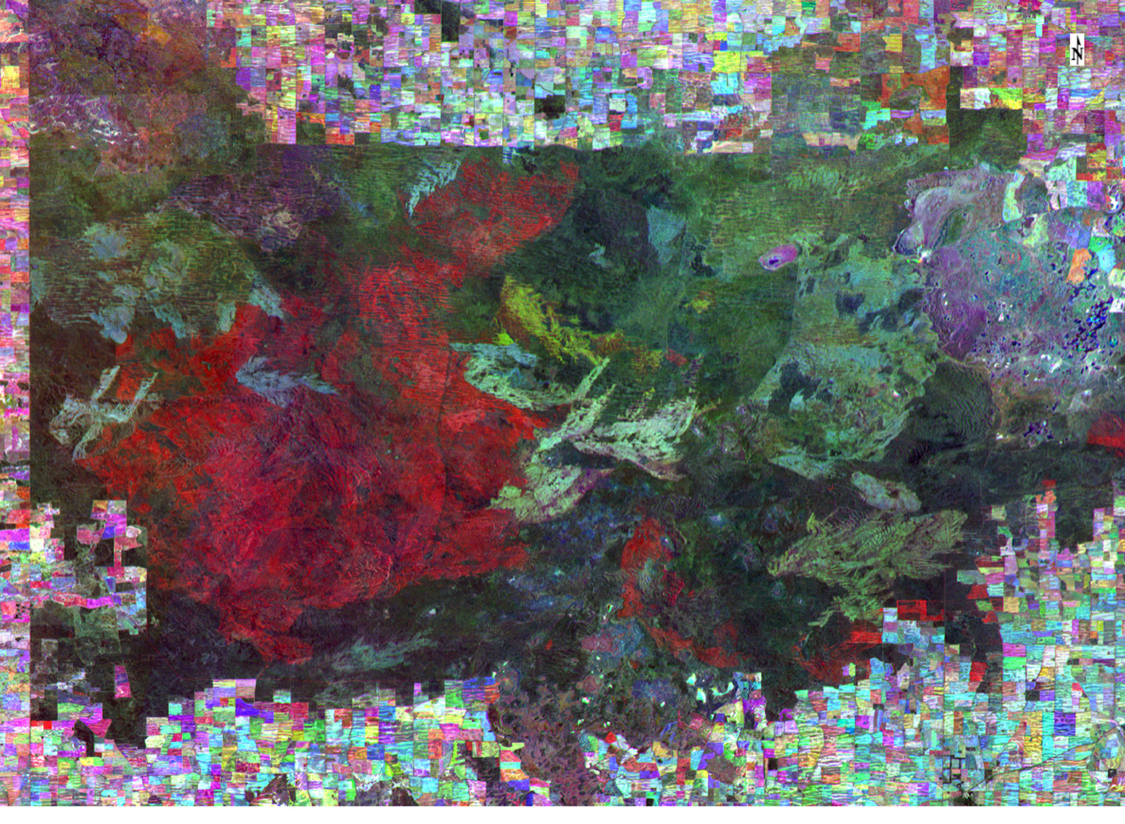
False colour composite Landsat MSS image from near-infrared (0.8–1.1 μm) bands of three images (1977, 1980 and 1985), highlighting fires in the Murray-Sunset National Park between 1977 to 1980 (red) and 1980 to 1985 (yellow). Older fire scars are also apparent in shades of blue, grey and green.

The modelling procedure builds on earlier analyses, including fire history mapping of the region [40] and predictive models of stem age based on stem diameter measurements [37]. Fire mapping was conducted using manual interpretation of Landsat satellite imagery, from 1972 to 2007 [40], plus additional mapping of fires from 2007 to 2011 undertaken using the same methods. Fig 1 shows how fires were highlighted by using a false colour composite image created from three Landsat near-infra red bands. In this example, band 7 of a 1977 and a 1980 image and band 4 of a 1985 image (0.8–1.1 μm) are used to create a false colour composite image. The fires observed in these images occurred in the same location as earlier more-generalised maps of fires in this location. The effect of wind direction on the fire front is apparent, and it is clear that this large scale change in the vegetation is due to fire. Using fire maps created from this technique, we found that 60% of mallee vegetation in the study area had not burnt between 1972 and 2011 [40].

To determine the age of sites burnt before 1972, data on stem diameters from sites burnt between 1972 and 2007 were modelled in relation to known fire age [37]. The diameter of all mallee stems was measured at each of 835 sites (485 with fire history calculated from fire maps [40], 350 known not have burnt since 1972). The mallee species was identified and mean annual rainfall for each site was obtained from the Bureau of Meteorology. The relationship between the mean stem diameter and time since fire was modelled separately for individuals of each of six species of mallee eucalypts by using generalised linear models (GLMs). The mean annual rainfall at each site was included as a second predictor variable (in addition to time-since-fire) to allow for the gradient in aridity.

To test the ability of these models to predict the age of mallee vegetation beyond the limits of current fire mapping, we collected additional data in February 2009 at ‘validation’ sites of known age >35 years since fire. We identified five areas where fires had occurred in 1917, 1932, 1951, 1957 and 1964, respectively, and sampled a total of 88 sites (at least 200 m apart). We compared the known age of these validation sites with the predictions generated from the GLMs of the mean stem diameter: this revealed a highly significant correlation between actual and predicted time since fire (r = 0.71, P < 0.001), thus confirming the utility of this method for ageing stands of mallee eucalypt vegetation. Extrapolation of these stem diameter models suggested that some sites may have escaped fire for more than 160 years [37].

Here, our aim was to produce a spatially explicit model identifying the age of areas that burned before 1972, by using sites where fire age was known or determined from stem diameter [37] as training data for a neural network model. The steps undertaken to create a spatio-temporal fire model were as follows.

1. Field surveys were conducted at sites where time-since-fire was known and the diameter of mallee stems was measured.
2. From these field data, the relationship between known time-since-fire and mallee stem diameter was modelled using linear regression, and tested on an independent data set [37].
3. These regression models were used to predict the fire age of sites of unknown time-since-fire.
4. At each of 622 points of fire age either known or determined from stem diameter, the site coordinates were used to extract the underlying pixel or polygon value for available data (including Landsat and radiometric bands, and vegetation type).
5. A predictive neural network model was developed to determine the relationship between the fire age of these known and determined sites, and Landsat, radiometric and vegetation data.
6. The neural network model was applied across the study area to create a detailed, regional map (25 x 25 m pixels) of predicted vegetation age post-fire.
7. The accuracy of the neural network model was assessed using independent validation data.

### Empirical data

Time-since-fire was determined at field sites from vegetation data collected from three surveys conducted between 2007 and 2010.

1. Vegetation assessments were conducted at 835 survey sites in July to August 2007 (see Clarke et al. [37] for details). Stem diameters and mallee eucalypt species were measured at each site from mallees located within 2 m either side of a 50 m transect. Site co-ordinates were recorded at the start of the transect. The year of the last fire (and hence time-since-fire) was determined by overlaying these points on fire history maps [40] for 485 of these sites; while the remaining 350 sites were known to be unburnt since 1972 (i.e. the earliest available Landsat imagery used in creating the fire history maps). The age of stems at these unburnt sites was determined from models of mallee stem diameters [37].
2. In February 2009, stem diameters were measured at 89 sites in Victoria and NSW where fires were known from management records to have occurred in 1917, 1932, 1951, 1957 and 1964 (i.e. 45–92 years since fire).
3. A further 80 sites were assessed in October to November 2010 where fires were known to have occurred between 1877 and 2006 (up to 130 years since fire) in South Australia and Victoria.

All sites burnt after 1977 were excluded to obtain the best model fit for older age classes. Sites burnt between 1972 and 1977 were retained, to maximise the number of sites of older age classes in the model. From these surveys, a total of 622 sites (279 from known fires or fire maps, 343 determined from stem diameter) that had last burnt between 1843 and 1977 were available to create a model of time-since-fire for mallee vegetation burnt prior to the availability of Landsat imagery.

### Modelling process

We used artificial neural networks (ANN) to model the relationship between fire age and a range of remotely sensed variables at the study sites (see below). ANN’s are a machine learning procedure which uses layers with interconnecting nodes (analogous to the human brain’s biological neurons) to model complex non-linear relationships between response variables and multiple predictor variables [47]. ANN’s are ideal for modelling complex ecological systems because they incorporate heterogeneous data, without needing to explicitly define underlying relationships [48]. The commonly used multilayer perceptron (MLP), the ANN method we employed, is outlined in more detail by Atkinson and Tatnall [47].

Our modelling approach included three steps: 1) a sensitivity analysis to select the remotely sensed variables to include in the model; 2) selected variables were entered into the neural network model to predict site age; 3) the model was used to create a predictive map of vegetation age across the study region.

Ninety-one environmental variables were considered for the modelling process, encompassing the following information: Landsat imagery, including between four Multispectral scanner (MSS) and seven Thematic Mapper (TM) and Enhanced Thematic Mapper (ETM) spectral bands for 15 different years (1972–2007: n = 81); Normalized Difference Vegetation Index (NDVI), representing vegetation ‘greenness’ (n = 1); the distribution of mallee vegetation classes (n = 3); radiometric data (n = 2); a Topographic Wetness Index, representing topographic and hydrologic processes (n = 3); and altitude (n = 1). Satellite imagery was acquired from Landsat Multi Spectral Scanner (1972, 1977, 1980, 1985 and 1988), Landsat Thematic Mapper (1989, 1991, 1992, 1995, 1998, 2004, 2005 and 2007) and Landsat Enhanced Thematic Mapper Plus (2000 and 2002). Pre-processing of these images included ortho-correction, radiometric correction, mosaicing of images, and calibration to a common geographic and spectral base (Australian Greenhouse Office year 2000 base [49]). These variables were extracted by recording the value of the underlying pixel or polygon from each digital map at the geographic coordinates of all 622 sites.

To avoid over-fitting of the model, the number of variables was reduced to 15 (including three classes of mallee vegetation) using the feature selection module in Statistica 10 (Table 1) [50]. The feature selection module calculates the ratio of the between-category variance to within-category variance (of the dependent variable) for categories or intervals (if continuous) of predictor variable. The sensitivity analysis ranks the variables by determining the resulting deterioration in the model if that variable was not included. When the sensitivity is one or lower, removing the variable has no negative effect on the predictive power of the model, or may enhance it [50]. See S1 Table for extracted Landsat and vegetation mallee class data at each survey point.

**Table 1:** The final set of input spatial layers selected for a neural network model to predict fire age, and the sensitivity of each variable.

Variable	Sensitivity
Mallee vegetation (3 classes)	1.72
1985 Band 3 Visible (0.63–0.69 μm)	1.12
1985 Band 4 Near-Infrared (0.76–0.90 μm)	1.07
2005 Band 5 Near-Infrared (1.55–1.75 μm)	1.05
2005 Band 1 Visible (0.45–0.52 μm)	1.05
2007 Band 3 Visible (0.63–0.69 μm)	1.04
2005 Band 2 Visible (0.52–0.60 μm)	1.04
2007 Band 5 Near-Infrared (1.55–1.75 μm)	1.03
2005 Band 7 Mid-Infrared (2.08–2.35 μm)	1.03
2007 Band 6 Thermal (10.40–12.50 μm)	1.01
2007 Band 7 Mid-Infrared (2.08–2.35 μm)	1.01
2007 Band 2 Visible (0.52–0.60 μm)	0.99
2007 Band 1 Visible (0.45–0.52 μm)	0.99

Neural network regression [50] was used to create 10 models of time-since-fire and the distribution of fire age, using varying numbers of hidden neurons and activation functions. The best performing model was selected based on r value, examination of the residuals and by excluding models that emphasised differences in mosaiced satellite images. A multilayer perceptron (MLP), neural network regression model with 15 continuous input variables, 10 hidden neurons and 1 output neuron was selected as the best performing model. It used a hyperbolic tangent (tanh) hidden activation function. This is a symmetric S-shaped (sigmoid) function that transforms the incoming signals from the neurons of the previous layer.

The modelling process used 60% of the training sites (of known and predicted ages) to build the model, 20% of sites to halt training and mitigate overfitting, and the remaining 20% of sites (which had no part in the model generation) to validate the model (Table 2). The model was then used to predict vegetation age at a resolution of 25 m. This pixel size was chosen as it was the resolution of the available Landsat imagery, it provided high resolution data for flora and fauna research and to avoid introduction of error through interpolation to a different pixel size. Areas where fires had been mapped as occurring between 1972 and 2011 were masked from the image, and analyses were not performed within these areas.

**Table 2:** Number of sites from each decade selected for model training, testing and validation.

Decade	Train	Test	Validation
1970–77	146	47	47
1960–69	35	16	15
1950–59	50	19	15
1940–49	34	12	9
1930–39	42	9	12
1920–29	14	9	7
1910–19	39	10	10
1900–09	5	0	5
1800s	9	2	4
**Total**	374	124	124

### Model validation

To assess the accuracy of the predicted vegetation age, we used a validation data set (i.e. data not used in building the remotely sensed model), which was comprised of 20% of the sites. To examine the relationship between the predicted ages of sites we performed a rank correlation (Spearman’s rho) between the predicted age of the validation sites (from the artificial neural network) and the age of sites known and determined from stem diameters. To further assess the accuracy of the remotely sensed model, we fitted a simple linear model in R v.3.1.1 [51] and calculated 95% prediction intervals. These intervals represent the region within which 95% of new data points (e.g. new predictions) will fall. This contrasts with a 95% confidence interval, which represents the region that 95% of samples would predict the true mean to fall within [52].

## Results

### Model validation

**Fig 2:**
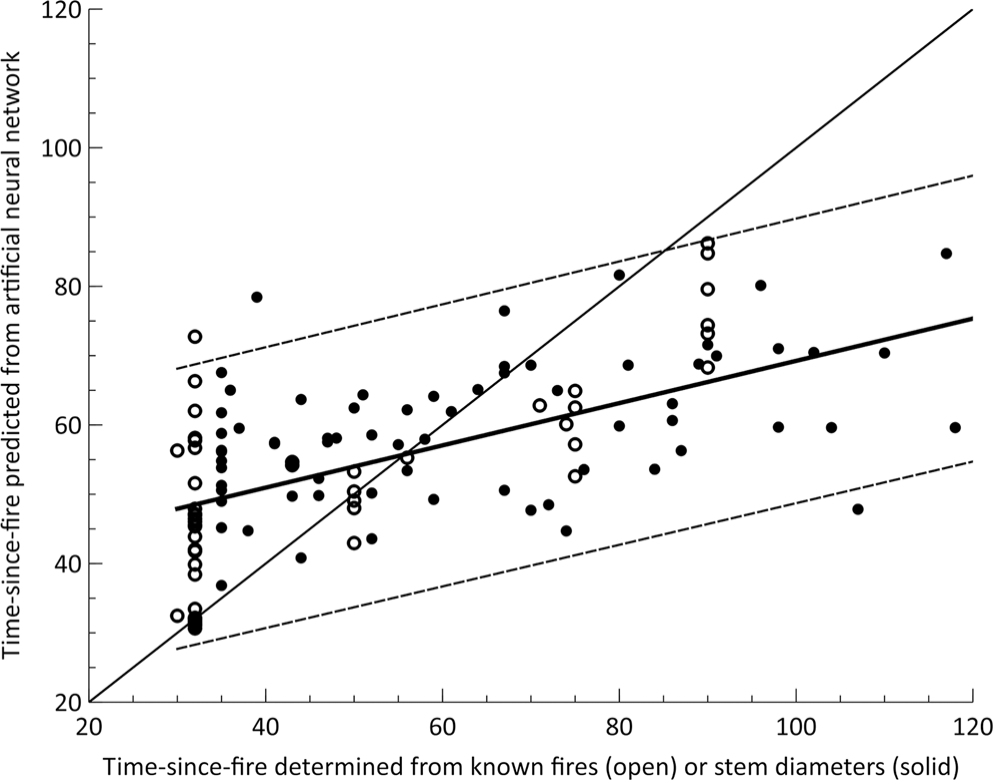
Relationship between the vegetation age at sites as determined from field data or of known age (x axis, predicted from stem diameter model [72 sites] and points of known fire history [52 sites]) and as predicted from remotely sensed data by using an ANN model. The mean is shown in bold, with 95% prediction errors. Also shown is the line of a 1:1 relationship.

The artificial neural network (ANN) model based on remotely sensed data provided good predictions of the post-fire ages of sites. For the 124 validation sites (sites not included in the model building process), the age estimated from the ANN model was strongly and positively related to the age predicted from field data and sites of known fire age (Spearman’s rho = 0.64; Fig 2). However, the ANN models had only limited capacity for identifying the age of very old sites (>90 years). For the validation sites, predictions of site age from the ANN models had a smaller range than age determined from stem diameters (30–88 years, compared with 30–164 years, respectively). Across the region, the maximum predicted age by the ANN model was 133 years since fire, whereas we are aware many sites are likely to be much older than this [37].

The resulting relationship between age predicted by the ANN model and by field data highlights a slope shallower than a 1:1 relationship, with the ANN model overestimating the age of younger sites and underestimating the age of older sites (Fig 2, estimate = 0.31, SE = 0.04). The 95% prediction errors generated from the linear model built using only the validation data highlights that for sites predicted to be <85 years since fire, the ANN model generally predicts vegetation age within 20 years of the age of sites from known fires and those determined by stem diameters (Fig 2).

### Map of vegetation age-classes

**Fig 3:**
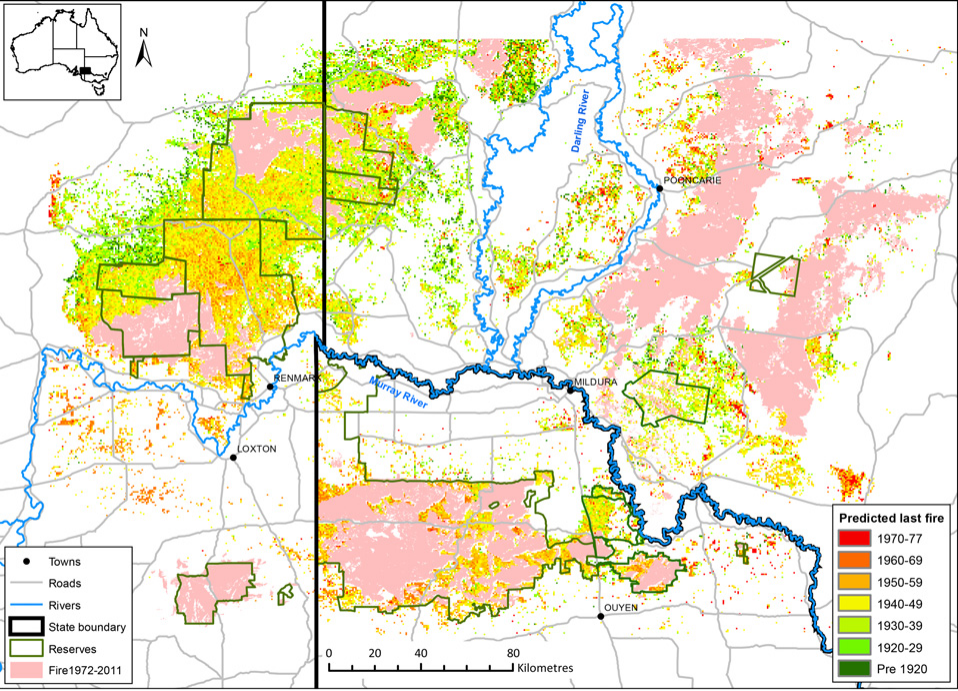
Map of the distribution of age-classes (attributed to fire) of mallee vegetation in the Murray Mallee region as predicted from the artificial neural network model.

Vegetation age was grouped into decades for mapping (Fig 3), with all ages over 90 years grouped into a single category (pre-1920). The oldest site mapped was predicted to be 133 years since fire; however, as Fig 3 illustrates, beyond 85 years, there was an increasing departure from the 1:1 line of perfect fit and under-estimation of age.

The mapped distribution of predicted age-classes of mallee vegetation (Fig 3) shows that vegetation age is not evenly distributed across the study region. To the south of the Murray River there is much less older mallee vegetation; vegetation greater than 60 years old is mainly in small fragmented blocks, and there is little vegetation older than 80 years. The largest block of older vegetation south of the Murray River is in Victoria in a large reserve (Hattah-Kulkyne National Park) in the south-east of the study area. Most very old mallee vegetation (i.e. > 80 years) is located north of the Murray River and is most extensive west of the Darling River system. Larger blocks of older vegetation also occur in this region and in South Australia along the north-west boundary of mallee vegetation in the study area. The southern and eastern parts of the region experienced large fires between 1972 to 2011 [40].

**Fig 4:**
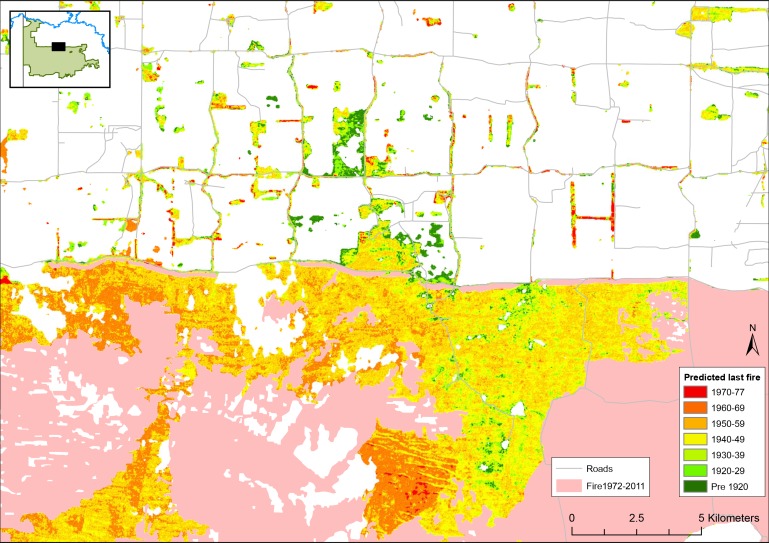
Zoomed in section of the map of the distribution of vegetation age-classes as predicted from the neural network model. This area in north-west Victoria shows the age classes of mallee vegetation along roadsides, and the east-west, dune-swale system.

The resolution of the predictive mapping (25 x 25 m pixels) allows some appreciation of the age of mallee vegetation along roadsides and in small remnant blocks on public and private land (Fig 4). Many roadsides support older mallee vegetation (i.e. > 60 years), particularly in Victoria where such older vegetation is scarce. At this resolution, the influence of the east-west, dune-swale system is also apparent on the mapped age of vegetation (Fig 4).

A boundary between satellite images is visible as a straight line in the north-west of the map (Fig 3). There is an apparent erroneous age difference of approximately 10 years to either side of this boundary in some places, due to variation between adjoining satellite images taken at different times within the same calendar year [49].

### Analysis of vegetation age-class distribution

**Fig 5:**
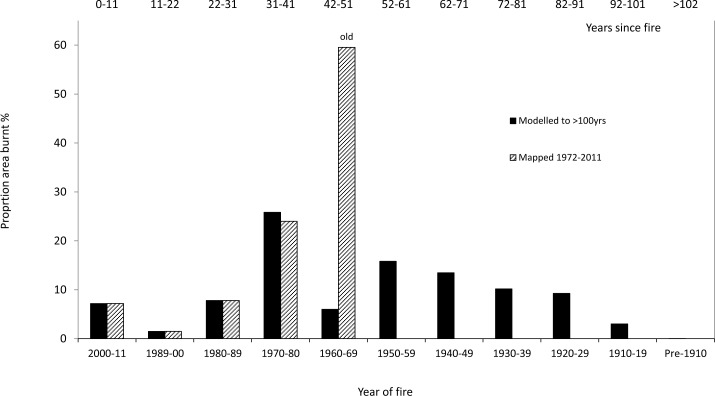
Age-class distribution of mallee stems comparing known fire history based on a) light shading—satellite imagery since 1972 (with all mallee vegetation greater than 1972 grouped together as ‘old’); and b) solid shading—a combination of mapping from 1972–2011 plus modelling of stem age from 1975 to pre-1900.

An analysis of the total area burnt during different time periods was undertaken from the mapped distribution, to illustrate the current age-class distribution of mallee vegetation in the region (Fig 5). Age-classes are depicted in two ways to highlight the gains from using the neural network model predictions. Method a) shows decadal classes from 1900 to 2011 based on the artificial neural network model of age-classes prior to 1972, combined with mapping of fires from Landsat imagery 1972 to 2011; and b) shows decadal classes from 1972 to 2011 based on Landsat mapping, with all older age-classes grouped as a single class labelled ‘old’. The artificial neural network model (method a) shows that over the last 80 years (1930–2011), 88% of mallee vegetation within the study region has burnt at least once. Using the latter method (b), 60% of mallee was classified as ‘old’ (Fig 5). The next largest peak in age-class is from the 1970s, due to extensive fires attributed to consecutive years of high rainfall in the early 1970s [46]. The artificial neural network model also suggests that a greater area burned each decade between the 1920s and1950s than has burned in the most recent three decades. Note that the peak in the 1970s does not necessarily imply that a greater area was burnt in this decade than prior decades, as the satellite imagery represents only what is present today. However, historical records [45,46,53–55] do not indicate any other such extensive fires since European settlement.

## Discussion

An ability to map the age of vegetation beyond the temporal limits of satellite imagery is of great benefit for the management of disturbance-prone systems, particularly those that display long-term successional dynamics. Here, by combining a technique for ageing mallee stems with a neural network model that uses remotely sensed data, we built a spatial model that maps the age-class distribution of vegetation across a region for age-classes of up to 100 years and more.

Succession in mallee vegetation remains dynamic for over a century [39,56]. As the distributions of many faunal species are closely associated with vegetation structure, faunal species are also influenced by fire over similarly long time-frames [57]. For example, species of reptile (e.g. Murray Striped Skink *Ctenotus brachyonyx*; [17]), bird (e.g. Yellow-plumed Honeyeater *Lichenostomus ornatus;* [42]) and mammal (e.g. Mallee Ningaui *Ningaui yvonneae*; [41]) prefer sites with vegetation that last burned at or beyond the limit of satellite imagery. Therefore, knowing the amount and spatial distribution of mallee vegetation of older age-classes is critical to the management of mallee biodiversity [42].

In addition to identifying *points* of high biodiversity value, a map also enables the overall extent of age classes to be calculated at various spatial scales such that managers can have a better understanding of vegetation age-structure in particular landscapes or conservation reserves. The spatial properties of fire mosaics, such as the spatial extent of different fire age-classes, are known to be key drivers of birds [12,58], small mammals [19] and reptiles [59] in this region. However, a limitation of these studies was that they were based on the properties of fire-mosaics only as described by satellite imagery, with an upper temporal bound of 34 years post-fire. It is likely that the spatial pattern of vegetation at various ages beyond 34 years post-fire, as mapped here, will also influence the fauna.

The level of correlation obtained during validation of the outputs of the neural network model suggest that there is room for improvement, potentially by incorporating other environmental factors that may contribute to the relationship between pixel reflectance in the satellite image and age of mallee vegetation. These could include the effects of grazing and browsing (including insect attack), rainfall, frost or windstorm.

### Distribution of age classes of mallee vegetation

The temporal pattern of age-classes in the Murray Mallee region (Fig 5) predominantly represents the extent of fire during each decade minus any subsequent fires. Pausas and Bradstock [60] describe a decrease in fire frequency in areas of lower productivity and lower rainfall to the north, compared with areas of higher productivity and fuel connectivity to the south of the study area. This may account for the greater occurrence of older mallee vegetation observed in the north of the study area.

The extent of fire was less during the second half of the 20^th^ century (with the exception of the 1970s), than in the 1920s to 1950s. A similar distribution of age classes was also found by Clarke et al [37]. As the model underestimates the age of older age-classes, some of the fires attributed to the 1920s to 1950s may represent fires that actually took place in earlier decades. However, the apparent greater extent of fire in the 1920s to 1950s could also be due to the use of fire for land clearing, whereby mallee vegetation was knocked down and allowed to dry before burning [45]. The region was being settled for grazing and cropping during this period and fire was also used for pasture improvement [61]. The extent to which these fires carried into surrounding mallee vegetation is not known, although early surveyor’s maps from the 1860s to 1930s include many references to burnt vegetation in the mallee region in Victoria (historical records from State Library Victoria and survey, parish and subdivision plans located at Land Victoria). Little is known of the pre-European fire regime and age-class distribution of vegetation in the Murray Mallee region [61].

The spike in area burnt in the early 1970s occurred when above-average rainfall produced abundant cover of spear grasses (*Austrostipa* spp.), which subsequently fuelled extensive fires across the region [46]. Weather conditions conducive to fire occur regularly in the region due to the hot, dry semi-arid climate. However, fuels are likely to be a limiting factor in many years [62]. Fuel loads in mallee vegetation accumulate relatively slowly with the cover of potential fuels peaking by approximately 20 years post-fire [39,62], and many fuel elements then decline around 90 to 100 years post-fire [39]. By reducing available fuel across nearly a third of the region, the large fires of 1974 and 1975 may be at least partially responsible for the apparent decrease in fire in subsequent decades. Avitabile et al. [40] showed that the current average inter-fire interval in the study region is likely to be much greater than 35 years, and that while the number of small fires has increased, the area burned in large fires has decreased since the 1970s. Successful fire management efforts (prescribed burning and fire suppression) may have resulted in fewer large fires in recent decades; however, there are insufficient data to verify this [40]. Many years of below-average rainfall in the region may also have contributed to low fuel abundance [40]. Extensive grazing can also reduce fuel loads [30]. Grazing pressure in national parks has declined in Victoria following herbivore control measures commencing in the 1980s, and the use of rabbit haemorrhagic disease in 1995 to control this introduced herbivore [27].

Only some 12% of the study region has escaped fire for more than 80 years. However, as the ANN model underestimates vegetation age, it is likely that these unburnt areas may be considerably older than the model predicts. Considering that lightning is the major source of ignition and occurs regularly across the region, and that large fires are relatively common, the observation that some areas have not burned suggests that they contain vegetation, structural, geographical, or bio-physical characteristics that make them less susceptible to fire. Fuels may also become more discontinuous after long periods without fire [39], and so areas which have survived 80 years without fire may have a higher probability of continuing to escape fire. This is consistent with some mallee eucalypts being found to survive over 160 years [37].

The method developed here has predicted the age-class structure up to 90 years prior to the latest satellite images used in modelling (2007), representing a significant advance in our ability to understand the implications of fire on ecosystem dynamics in the region.

### Future directions for research

The modelling technique described here provides a snapshot of the current age-classes of vegetation within the study area. Each pixel on the map is assigned the year of the most recent stand-replacing disturbance (in this case, most commonly fire). Currently, this approach does not distinguish areas that have burnt multiple times, which could display different biological characteristics as a result of differing fire history; and it tends to underestimate the vegetation age for the oldest sites (> 85 years). The models could be improved with further work to identify processes that drive change with increasing time; for example, localised mallee death due to severe weather conditions (drought, wind storm, frost). Recruitment events between fires could also introduce uncertainty into the model, although these appear to be extremely rare [63]).

### Broader use of these techniques

The approach described here has potential for use in determining age classes of other vegetation types. It could be used to map the temporal distribution of stand ages following any stand-replacing disturbance, including fire, cyclones, landslides or slash and burn agriculture. However, the following assumptions are likely to be necessary for this approach to be successful:

1. an ability to reliably age sufficient sites across the study area to train the model
2. reliable and consistent recruitment of overstorey following stand-replacing disturbance
3. minimal recruitment of overstorey between disturbance events
4. spectrally distinct changes occurring in the vegetation over time, such that differences in satellite imagery can be detected.

Patterns of change in structural characteristics of mallee vegetation over time have been described [39], and are expected to be closely related to spectral attributes, such as increased reflectance due to bare ground and changes in infrared reflectance due to change in vegetation cover. Similarly, changes in reflectance in other forest types have been linked to changes in chlorophyll content as trees age and the density of stems declines over time [9].

## Conclusion

This study outlines a method to determine a snapshot of the current distribution of vegetation age-classes in the fire-prone Murray Mallee region over an ecologically meaningful time span, and provides valuable information on the likely fire history of the region. The occurrence of very old mallee vegetation on roadsides and in other small pockets highlights the need for appropriate management of this unevenly distributed, and in places, rare resource. It is likely that these small patches retain important habitat components such as hollows, particularly in the south of the study area where long-unburnt vegetation is scarce. The uneven distribution of age classes suggests that managing mallee vegetation in a coordinated way across state and reserve boundaries will be beneficial. Achieving a desirable distribution of age classes within individual reserves needs to be balanced with maintaining age-class structure and diversity across the entire region.

This model does not elucidate the ‘natural’ (pre-settlement) fire regime, nor is it likely that a return to such a regime would be helpful in today’s highly fragmented and modified landscapes. While it is clear that large homogenising fires should be avoided to preserve a diversity of habitats, both within reserves and across the region, identifying spatial and temporal configurations of age classes across the landscape that are more, or less, desirable for achieving conservation goals remains an urgent challenge.

## Supporting Information

S1 Table Landsat bands and vegetation mallee class data used for creating artificial neural network models. (DOCX)
