# Supplementary material for: Historical Maps from Modern Images: Using Remote Sensing to Model and Map Century-Long Vegetation Change in a Fire-Prone Region
Source: PLoS One. 2016 Mar 30;11(3):e0150808. doi: 10.1371/journal.pone.0150808 (PMC4814043; doi:10.1371/journal.pone.0150808)
Supplement: S1 Table — (DOCX) [file pone.0150808.s001.docx]

**S1 Table. Landsat bands and vegetation mallee class data used for creating artificial neural network models.**

(Vegetation class; 1=Triodia Mallee, 2=Chenopod Mallee, 3=Heathy Mallee)

| Site ID | Easting | Northing | Fire year | B6_2007 | B7_2007 | B5_2007 | B1_2007 | B2_2005 | Mallee Vegetation Class | B1_2005 | B4_1985 | B5_2005 | B3_2007 | B2_2007 | B3_1985 |
| --- | --- | --- | --- | --- | --- | --- | --- | --- | --- | --- | --- | --- | --- | --- | --- |
| B07S04 | 560263 | 6158530 | 1843 | 157.0 | 84.4 | 144.1 | 81.9 | 36.0 | 1 | 41.3 | 54.9 | 118.8 | 62.9 | 43.6 | 52.9 |
| B11S03 | 508030 | 6159047 | 1845 | 148.0 | 64.2 | 120.9 | 67.6 | 31.2 | 2 | 42.2 | 58.4 | 109.5 | 43.1 | 32.2 | 56.5 |
| M21S07 | 504164 | 6339073 | 1853 | 156.6 | 77.1 | 134.3 | 73.1 | 34.1 | 2 | 43.0 | 56.6 | 117.1 | 55.5 | 37.1 | 58.1 |
| B16S15 | 412535 | 6269988 | 1869 | 151.0 | 79.1 | 137.1 | 78.5 | 35.8 | 2 | 38.9 | 61.3 | 123.9 | 68.9 | 43.6 | 62.6 |
| CMO01 | 428114 | 6271931 | 1877 | 153.0 | 73.5 | 131.9 | 73.3 | 34.2 | 2 | 38.1 | 50.8 | 122.4 | 53.8 | 37.2 | 52.8 |
| B20S15 | 490488 | 6338987 | 1879 | 155.0 | 88.7 | 144.7 | 72.0 | 33.0 | 2 | 43.0 | 54.0 | 119.9 | 60.8 | 37.9 | 53.7 |
| CMO32 | 641516 | 6143573 | 1882 | 159.0 | 66.0 | 129.6 | 77.6 | 25.1 | 2 | 32.7 | 49.1 | 90.8 | 48.9 | 37.1 | 49.9 |
| M05S08 | 568613 | 6172735 | 1884 | 156.9 | 62.9 | 119.6 | 71.6 | 26.3 | 2 | 35.1 | 55.2 | 94.0 | 45.3 | 33.8 | 53.7 |
| M08S04 | 545736 | 6175988 | 1889 | 154.0 | 68.7 | 122.4 | 71.6 | 36.0 | 1 | 46.0 | 58.7 | 111.1 | 48.7 | 35.9 | 56.6 |
| B05S16 | 569010 | 6173996 | 1890 | 158.0 | 70.6 | 127.5 | 73.3 | 30.6 | 2 | 40.6 | 60.4 | 107.5 | 49.8 | 36.8 | 58.1 |
| B16S13 | 413021 | 6269524 | 1890 | 151.0 | 96.2 | 156.2 | 79.1 | 38.7 | 2 | 42.4 | 62.8 | 131.8 | 68.8 | 43.7 | 64.1 |
| CMO09 | 413972 | 6272040 | 1890 | 152.0 | 82.7 | 141.0 | 72.9 | 37.3 | 2 | 38.7 | 66.4 | 134.0 | 62.6 | 40.7 | 64.2 |
| B24S20 | 508385 | 6309525 | 1892 | 154.0 | 79.3 | 137.9 | 71.7 | 37.0 | 1 | 45.4 | 56.8 | 125.2 | 60.6 | 38.6 | 58.6 |
| B06S13 | 569533 | 6159490 | 1897 | 159.0 | 78.2 | 137.5 | 78.1 | 31.1 | 1 | 40.6 | 56.0 | 110.5 | 55.0 | 39.3 | 54.2 |
| B05S17 | 568520 | 6173989 | 1899 | 157.0 | 60.6 | 118.5 | 70.3 | 27.7 | 2 | 36.0 | 50.7 | 98.6 | 44.2 | 32.2 | 50.3 |
| B03S06 | 590231 | 6153500 | 1900 | 153.0 | 64.2 | 119.3 | 75.5 | 29.7 | 2 | 40.2 | 57.4 | 101.6 | 49.1 | 37.4 | 54.4 |
| B20S05 | 490500 | 6337532 | 1901 | 156.0 | 81.5 | 135.6 | 70.3 | 30.5 | 1 | 38.5 | 54.6 | 118.3 | 56.6 | 36.9 | 52.9 |
| B01S19 | 616979 | 6165008 | 1902 | 155.2 | 79.6 | 137.8 | 80.1 | 35.5 | 1 | 45.1 | 56.0 | 114.8 | 60.4 | 43.6 | 54.4 |
| M16S06 | 411671 | 6269534 | 1902 | 149.0 | 74.1 | 127.3 | 72.4 | 32.4 | 2 | 37.4 | 63.2 | 108.1 | 54.1 | 36.9 | 62.5 |
| B26S12 | 647615 | 6210534 | 1903 | 158.0 | 62.8 | 114.1 | 69.9 | 28.0 | 2 | 36.1 | 58.1 | 101.7 | 45.4 | 33.3 | 56.1 |
| B16S20 | 411511 | 6269473 | 1903 | 149.0 | 74.7 | 130.5 | 74.5 | 33.3 | 2 | 37.0 | 64.0 | 112.0 | 58.8 | 39.5 | 65.0 |
| B01S08 | 617518 | 6167024 | 1905 | 157.0 | 69.3 | 123.6 | 73.4 | 28.1 | 1 | 35.7 | 55.1 | 92.2 | 50.5 | 37.2 | 54.5 |
| M01S01 | 616213 | 6166917 | 1905 | 157.0 | 77.7 | 135.0 | 78.5 | 34.6 | 1 | 42.0 | 61.0 | 111.3 | 59.3 | 42.0 | 60.2 |
| B23S13 | 514272 | 6304493 | 1906 | 152.1 | 69.5 | 122.8 | 71.9 | 33.1 | 1 | 45.2 | 53.8 | 111.2 | 54.9 | 37.0 | 57.2 |
| B01S03 | 615517 | 6166481 | 1907 | 157.0 | 66.6 | 120.4 | 72.3 | 28.6 | 1 | 37.0 | 52.6 | 98.7 | 47.3 | 35.3 | 53.1 |
| B06S12 | 569030 | 6159497 | 1907 | 157.0 | 89.0 | 148.8 | 84.9 | 37.0 | 1 | 44.5 | 56.6 | 119.5 | 67.1 | 46.9 | 53.2 |
| M01S02 | 616433 | 6166884 | 1908 | 156.9 | 89.3 | 147.4 | 81.6 | 36.4 | 1 | 44.5 | 58.2 | 116.9 | 66.3 | 45.7 | 61.6 |
| CMO23 | 571562 | 6164564 | 1908 | 156.0 | 64.1 | 118.1 | 71.0 | 27.1 | 2 | 36.0 | 57.3 | 92.3 | 40.6 | 32.2 | 53.8 |
| B02S08 | 608007 | 6150974 | 1909 | 154.8 | 73.3 | 130.4 | 78.6 | 30.9 | 1 | 40.8 | 55.1 | 102.1 | 54.2 | 39.7 | 53.9 |
| B23S19 | 515368 | 6304489 | 1909 | 153.7 | 84.3 | 142.3 | 71.4 | 36.5 | 2 | 45.8 | 62.6 | 121.2 | 62.7 | 40.7 | 61.0 |
| CMO11 | 411636 | 6268106 | 1909 | 146.2 | 70.7 | 125.3 | 74.1 | 33.8 | 2 | 36.1 | 62.5 | 109.9 | 54.4 | 38.1 | 61.4 |
| B26S13 | 647545 | 6211036 | 1910 | 159.0 | 67.6 | 121.3 | 69.0 | 27.2 | 2 | 32.8 | 58.4 | 100.0 | 46.0 | 33.0 | 56.6 |
| M06S01 | 567072 | 6159098 | 1910 | 157.0 | 74.0 | 133.0 | 71.0 | 28.5 | 2 | 35.1 | 52.0 | 107.7 | 50.0 | 36.0 | 50.8 |
| B21S14 | 501520 | 6338895 | 1910 | 158.0 | 80.4 | 139.0 | 72.7 | 33.9 | 2 | 42.9 | 59.2 | 126.2 | 57.0 | 38.0 | 60.8 |
| M06S09 | 569379 | 6158317 | 1911 | 158.0 | 81.1 | 140.2 | 81.1 | 33.8 | 1 | 42.9 | 54.1 | 116.5 | 59.4 | 41.3 | 53.1 |
| B24S14 | 508005 | 6310054 | 1911 | 155.7 | 82.9 | 143.5 | 71.2 | 33.4 | 1 | 43.9 | 58.7 | 122.1 | 56.9 | 36.6 | 62.6 |
| CMO29 | 572921 | 6164514 | 1911 | 155.1 | 61.8 | 118.8 | 70.4 | 28.5 | 2 | 35.5 | 56.4 | 102.5 | 46.6 | 34.2 | 53.9 |
| M16S04 | 411662 | 6268978 | 1912 | 150.4 | 72.9 | 127.0 | 74.9 | 33.3 | 2 | 39.3 | 62.2 | 117.3 | 56.8 | 38.7 | 64.3 |
| M04S09 | 589081 | 6145750 | 1913 | 154.3 | 58.3 | 113.2 | 70.0 | 26.7 | 2 | 35.4 | 50.5 | 93.6 | 40.7 | 31.0 | 48.7 |
| B01S09 | 617003 | 6167032 | 1913 | 156.7 | 71.3 | 127.3 | 71.6 | 28.2 | 1 | 35.2 | 54.1 | 101.0 | 48.4 | 35.5 | 52.2 |
| B04S08 | 587973 | 6145505 | 1915 | 148.7 | 59.3 | 107.1 | 72.3 | 28.0 | 1 | 36.3 | 37.5 | 86.0 | 43.1 | 34.4 | 39.6 |
| B02S16 | 609039 | 6150512 | 1916 | 154.0 | 58.3 | 103.6 | 73.9 | 28.2 | 1 | 40.8 | 52.0 | 89.2 | 44.2 | 35.1 | 50.9 |
| B03S20 | 591518 | 6154982 | 1916 | 153.0 | 61.7 | 112.3 | 75.4 | 30.0 | 1 | 39.0 | 47.5 | 88.5 | 46.8 | 36.8 | 48.4 |
| M05S07 | 568964 | 6173032 | 1916 | 157.8 | 87.5 | 149.0 | 80.7 | 36.1 | 2 | 45.5 | 61.9 | 129.0 | 65.1 | 43.7 | 59.9 |
| B23S03 | 515985 | 6307000 | 1916 | 154.0 | 88.2 | 147.6 | 75.6 | 37.6 | 1 | 46.5 | 58.0 | 122.3 | 65.8 | 42.2 | 60.6 |
| B02S09 | 608027 | 6151504 | 1917 | 157.0 | 74.5 | 131.9 | 79.7 | 34.7 | 1 | 45.6 | 59.6 | 113.9 | 57.1 | 41.4 | 60.7 |
| M01S04 | 616177 | 6166509 | 1917 | 156.6 | 77.6 | 134.5 | 78.6 | 33.9 | 1 | 43.0 | 57.6 | 108.6 | 58.3 | 41.6 | 56.6 |
| B01S02 | 615372 | 6167035 | 1917 | 157.0 | 76.8 | 133.8 | 76.6 | 31.5 | 1 | 39.2 | 56.5 | 106.3 | 56.4 | 39.7 | 56.5 |
| M02S03 | 609736 | 6152150 | 1917 | 158.0 | 88.6 | 149.7 | 81.2 | 35.5 | 1 | 44.1 | 61.3 | 116.6 | 63.3 | 43.2 | 61.2 |
| 1917_28 | 527107 | 6334686 | 1917 | 155.0 | 89.9 | 148.4 | 74.2 | 37.3 | 1 | 46.6 | 77.6 | 115.7 | 64.3 | 42.9 | 77.5 |
| 1917_3 | 519980 | 6292787 | 1917 | 155.0 | 74.6 | 131.6 | 67.3 | 35.1 | 1 | 42.7 | 65.0 | 117.3 | 55.1 | 36.3 | 64.0 |
| 1917_25 | 526662 | 6336985 | 1917 | 155.0 | 74.0 | 132.1 | 73.0 | 36.9 | 2 | 44.0 | 66.8 | 116.5 | 59.3 | 39.0 | 66.1 |
| 1917_7 | 516765 | 6296083 | 1917 | 152.2 | 72.5 | 124.3 | 72.6 | 31.8 | 1 | 42.3 | 56.0 | 102.1 | 59.0 | 37.7 | 56.9 |
| 1917_29 | 527016 | 6334911 | 1917 | 156.0 | 79.4 | 134.3 | 74.1 | 37.9 | 1 | 46.1 | 66.0 | 122.7 | 60.0 | 38.5 | 69.3 |
| 1917_4 | 518803 | 6293989 | 1917 | 152.1 | 73.3 | 130.3 | 71.0 | 38.8 | 1 | 47.8 | 68.9 | 124.2 | 59.2 | 38.5 | 66.8 |
| 1917_18 | 514251 | 6310519 | 1917 | 154.0 | 83.7 | 145.8 | 72.9 | 37.3 | 2 | 45.4 | 59.6 | 119.0 | 63.0 | 40.8 | 58.1 |
| 1917_19 | 513967 | 6310604 | 1917 | 154.0 | 70.8 | 128.3 | 71.1 | 31.5 | 1 | 40.1 | 56.0 | 105.9 | 53.8 | 35.2 | 54.6 |
| 1917_26 | 526842 | 6335903 | 1917 | 157.8 | 82.9 | 142.7 | 72.5 | 32.9 | 1 | 41.1 | 63.1 | 115.8 | 57.6 | 38.4 | 63.5 |
| 1917_8 | 516395 | 6296470 | 1917 | 153.0 | 74.6 | 130.2 | 72.2 | 33.9 | 1 | 45.4 | 60.3 | 116.7 | 57.8 | 38.3 | 62.2 |
| 1917_9 | 516244 | 6296603 | 1917 | 152.0 | 81.4 | 137.2 | 71.7 | 33.1 | 1 | 44.4 | 57.1 | 106.3 | 62.1 | 39.2 | 58.8 |
| 1917_30 | 527303 | 6334404 | 1917 | 155.3 | 77.4 | 138.4 | 73.1 | 35.8 | 1 | 44.9 | 67.0 | 123.4 | 59.2 | 39.3 | 64.6 |
| 1917_22 | 526769 | 6337308 | 1917 | 157.0 | 83.1 | 143.1 | 73.2 | 33.0 | 1 | 43.1 | 64.2 | 117.9 | 61.2 | 39.1 | 63.1 |
| 1917_1 | 520443 | 6292397 | 1917 | 151.2 | 83.6 | 140.6 | 71.2 | 33.5 | 1 | 44.2 | 64.2 | 113.4 | 64.9 | 39.8 | 63.9 |
| 1917_21 | 520829 | 6309749 | 1917 | 156.5 | 69.8 | 128.1 | 68.7 | 35.3 | 2 | 41.8 | 61.8 | 121.6 | 50.2 | 34.4 | 59.6 |
| 1917_2 | 520191 | 6292649 | 1917 | 152.0 | 81.6 | 137.8 | 72.4 | 33.0 | 1 | 44.5 | 63.0 | 115.2 | 63.0 | 38.9 | 62.2 |
| 1917_5 | 518670 | 6294178 | 1917 | 153.0 | 82.0 | 140.4 | 73.5 | 33.1 | 1 | 43.7 | 55.4 | 109.7 | 62.3 | 39.0 | 56.5 |
| 1917_16 | 506152 | 6311520 | 1917 | 154.3 | 79.1 | 129.2 | 70.9 | 35.2 | 1 | 44.1 | 55.2 | 112.7 | 56.9 | 37.1 | 59.8 |
| 1917_13 | 505210 | 6311646 | 1917 | 152.7 | 77.9 | 126.5 | 71.2 | 37.0 | 1 | 46.5 | 58.8 | 123.5 | 59.6 | 39.4 | 61.5 |
| 1917_6 | 517269 | 6295560 | 1917 | 153.9 | 80.9 | 146.2 | 70.8 | 34.9 | 1 | 43.2 | 65.0 | 113.6 | 65.2 | 41.0 | 63.5 |
| 1917_24 | 526647 | 6336630 | 1917 | 154.8 | 77.7 | 134.3 | 70.7 | 35.0 | 2 | 40.6 | 66.3 | 119.7 | 58.7 | 37.9 | 66.5 |
| 1917_23 | 526811 | 6337893 | 1917 | 155.0 | 81.7 | 139.9 | 73.8 | 35.8 | 1 | 42.0 | 63.7 | 123.4 | 64.7 | 40.7 | 66.3 |
| 1917_11 | 504464 | 6311678 | 1917 | 154.0 | 89.3 | 147.3 | 76.7 | 38.2 | 1 | 47.9 | 55.4 | 128.9 | 71.5 | 42.5 | 58.2 |
| 1917_27 | 526723 | 6336187 | 1917 | 156.0 | 97.2 | 163.2 | 71.8 | 34.2 | 2 | 44.6 | 70.3 | 123.0 | 67.0 | 41.5 | 67.6 |
| 1917_12 | 504123 | 6311729 | 1917 | 153.7 | 88.2 | 146.0 | 73.4 | 37.9 | 1 | 46.8 | 56.7 | 126.2 | 65.3 | 39.1 | 59.4 |
| 1917_15 | 506535 | 6311481 | 1917 | 154.3 | 79.7 | 129.4 | 73.2 | 37.3 | 1 | 44.2 | 56.3 | 130.9 | 59.3 | 38.8 | 57.9 |
| 1917_14 | 504839 | 6311643 | 1917 | 154.0 | 92.9 | 152.4 | 75.6 | 39.4 | 1 | 47.5 | 57.2 | 134.9 | 69.4 | 41.6 | 60.1 |
| M03S02 | 591060 | 6155412 | 1918 | 153.0 | 66.8 | 117.2 | 76.1 | 29.0 | 1 | 39.1 | 51.6 | 95.8 | 49.0 | 38.1 | 49.6 |
| M21S10 | 503151 | 6339883 | 1918 | 157.0 | 81.8 | 139.6 | 71.0 | 31.7 | 2 | 42.9 | 52.8 | 114.9 | 56.3 | 37.3 | 54.0 |
| B24S08 | 510501 | 6309943 | 1918 | 156.1 | 85.6 | 143.6 | 71.7 | 35.9 | 1 | 45.0 | 63.9 | 127.8 | 62.0 | 38.4 | 66.7 |
| B03S18 | 591522 | 6154021 | 1919 | 154.0 | 71.0 | 127.0 | 77.3 | 31.5 | 1 | 41.5 | 49.5 | 105.1 | 51.3 | 38.7 | 51.4 |
| B06S03 | 567968 | 6160005 | 1919 | 158.0 | 74.0 | 131.0 | 78.0 | 32.1 | 1 | 43.0 | 51.6 | 107.5 | 53.0 | 39.0 | 50.3 |
| B16S14 | 413001 | 6270027 | 1919 | 151.0 | 78.9 | 141.0 | 75.8 | 34.5 | 2 | 39.5 | 62.8 | 119.7 | 62.4 | 39.7 | 62.0 |
| B24S12 | 508476 | 6310537 | 1919 | 154.0 | 86.4 | 142.4 | 70.6 | 33.4 | 1 | 42.4 | 57.8 | 120.2 | 60.5 | 38.0 | 57.9 |
| CMO03 | 427832 | 6272185 | 1919 | 150.0 | 64.3 | 120.0 | 72.5 | 27.8 | 2 | 33.2 | 53.1 | 102.4 | 47.7 | 34.8 | 54.3 |
| B04S01 | 589490 | 6146608 | 1920 | 151.0 | 62.9 | 111.7 | 72.5 | 29.4 | 3 | 38.1 | 40.5 | 93.3 | 44.9 | 36.0 | 42.1 |
| B02S13 | 609519 | 6151485 | 1920 | 158.0 | 83.0 | 145.2 | 80.7 | 35.8 | 1 | 43.8 | 60.2 | 120.8 | 62.4 | 42.7 | 59.0 |
| B14S02 | 426425 | 6260519 | 1921 | 150.3 | 68.8 | 126.2 | 74.7 | 29.4 | 2 | 34.1 | 54.8 | 98.4 | 52.2 | 37.7 | 56.2 |
| M19S06 | 483635 | 6334287 | 1921 | 156.0 | 69.9 | 122.9 | 71.0 | 32.8 | 1 | 44.8 | 48.2 | 107.2 | 50.2 | 35.3 | 52.1 |
| B03S13 | 592511 | 6154479 | 1922 | 155.0 | 81.1 | 142.5 | 86.1 | 38.1 | 1 | 49.2 | 59.0 | 118.2 | 61.5 | 45.9 | 56.5 |
| B01S06 | 616983 | 6166509 | 1923 | 155.0 | 53.3 | 103.7 | 66.9 | 25.4 | 1 | 32.1 | 56.6 | 85.7 | 36.6 | 29.6 | 52.9 |
| B02S14 | 609478 | 6150996 | 1923 | 155.4 | 58.4 | 110.9 | 69.8 | 28.6 | 1 | 38.2 | 51.2 | 92.8 | 42.6 | 33.4 | 51.2 |
| M07S04 | 562269 | 6158504 | 1923 | 155.0 | 56.0 | 109.0 | 69.0 | 26.3 | 2 | 35.2 | 53.0 | 92.4 | 42.0 | 34.0 | 53.6 |
| M06S04 | 567669 | 6159128 | 1923 | 156.4 | 69.9 | 125.8 | 77.5 | 30.7 | 2 | 40.0 | 50.4 | 108.1 | 52.5 | 38.5 | 48.1 |
| M03S03 | 590313 | 6154998 | 1923 | 150.4 | 66.2 | 115.0 | 77.0 | 29.6 | 1 | 40.2 | 46.7 | 93.1 | 50.3 | 39.2 | 49.7 |
| B02S11 | 609517 | 6152486 | 1923 | 156.0 | 78.9 | 130.9 | 80.9 | 35.0 | 1 | 45.0 | 63.0 | 104.7 | 56.0 | 40.9 | 65.6 |
| B04S04 | 589480 | 6145990 | 1923 | 152.0 | 70.8 | 130.2 | 77.1 | 31.8 | 1 | 42.6 | 51.5 | 104.0 | 50.2 | 37.9 | 52.1 |
| B26S16 | 646481 | 6211422 | 1923 | 160.0 | 67.0 | 121.9 | 68.5 | 26.7 | 2 | 36.3 | 52.8 | 98.5 | 45.7 | 33.6 | 50.7 |
| M06S02 | 567048 | 6158586 | 1923 | 157.0 | 72.3 | 132.2 | 74.8 | 29.5 | 2 | 36.5 | 51.9 | 108.9 | 51.9 | 37.5 | 50.0 |
| CMO06 | 420949 | 6271288 | 1923 | 150.0 | 61.5 | 116.6 | 71.4 | 28.4 | 2 | 33.5 | 63.8 | 93.5 | 47.5 | 34.5 | 62.8 |
| B03S19 | 591492 | 6154528 | 1925 | 152.9 | 70.1 | 122.5 | 77.8 | 31.7 | 1 | 40.2 | 52.2 | 98.2 | 52.0 | 39.0 | 52.7 |
| M02S06 | 607068 | 6152085 | 1926 | 154.8 | 60.8 | 111.7 | 68.2 | 27.8 | 1 | 35.1 | 68.1 | 97.4 | 44.0 | 32.8 | 66.0 |
| M05S04 | 569496 | 6174736 | 1926 | 156.0 | 56.7 | 113.0 | 68.4 | 26.4 | 2 | 34.4 | 50.0 | 92.5 | 39.0 | 30.7 | 47.4 |
| M21S08 | 504013 | 6339226 | 1926 | 157.0 | 81.0 | 134.9 | 73.6 | 37.2 | 2 | 45.6 | 56.9 | 125.4 | 57.3 | 38.3 | 58.4 |
| B01S13 | 615492 | 6165509 | 1926 | 157.0 | 79.0 | 138.8 | 78.3 | 36.7 | 1 | 43.8 | 54.3 | 115.6 | 60.7 | 42.6 | 58.4 |
| M21S09 | 503524 | 6339458 | 1926 | 156.4 | 87.8 | 146.3 | 74.1 | 37.0 | 2 | 45.5 | 62.2 | 124.7 | 60.9 | 40.0 | 63.4 |
| B11S01 | 507994 | 6159990 | 1927 | 150.0 | 53.4 | 104.2 | 66.6 | 27.4 | 2 | 38.5 | 54.7 | 81.0 | 38.6 | 31.7 | 52.7 |
| B16S02 | 411002 | 6268023 | 1927 | 147.0 | 67.4 | 121.2 | 73.1 | 31.3 | 2 | 35.5 | 60.3 | 103.0 | 51.9 | 37.0 | 59.7 |
| B24S16 | 507500 | 6309490 | 1927 | 154.0 | 84.8 | 147.0 | 73.2 | 38.1 | 2 | 44.6 | 65.9 | 126.6 | 62.6 | 39.4 | 65.0 |
| B06S02 | 567523 | 6159988 | 1928 | 156.0 | 71.0 | 126.0 | 76.0 | 31.0 | 1 | 38.0 | 50.1 | 99.1 | 52.0 | 38.0 | 51.8 |
| B23S12 | 514237 | 6304017 | 1928 | 154.0 | 69.4 | 124.8 | 69.0 | 30.0 | 1 | 40.4 | 53.6 | 99.0 | 51.0 | 35.9 | 51.3 |
| CMO19 | 411680 | 6262886 | 1928 | 150.0 | 60.1 | 115.4 | 72.7 | 27.0 | 2 | 31.7 | 59.6 | 93.3 | 44.8 | 34.9 | 58.4 |
| B03S12 | 592029 | 6154486 | 1929 | 153.0 | 66.5 | 120.5 | 78.2 | 30.6 | 1 | 41.2 | 47.9 | 98.4 | 50.2 | 38.9 | 50.0 |
| B21S13 | 501968 | 6338946 | 1929 | 155.0 | 70.7 | 126.4 | 69.1 | 33.7 | 2 | 42.7 | 60.3 | 118.4 | 49.1 | 35.1 | 60.0 |
| B01S05 | 616480 | 6166512 | 1929 | 157.0 | 85.2 | 143.7 | 79.3 | 33.0 | 1 | 42.7 | 59.0 | 109.0 | 59.8 | 42.8 | 60.5 |
| M02S05 | 607104 | 6152323 | 1930 | 154.0 | 66.3 | 119.2 | 74.3 | 29.7 | 1 | 39.0 | 57.2 | 101.1 | 47.8 | 36.2 | 61.5 |
| B23S18 | 514771 | 6304500 | 1930 | 153.0 | 73.0 | 128.4 | 72.0 | 32.3 | 1 | 44.0 | 56.6 | 112.7 | 55.5 | 36.8 | 58.4 |
| B04S10 | 588490 | 6145987 | 1931 | 148.0 | 60.0 | 108.5 | 76.5 | 29.8 | 3 | 39.9 | 34.9 | 91.4 | 45.5 | 35.6 | 39.5 |
| M01S07 | 615465 | 6165211 | 1931 | 156.0 | 65.2 | 120.0 | 69.7 | 28.0 | 1 | 36.8 | 53.5 | 95.7 | 44.7 | 34.7 | 51.0 |
| B06S04 | 568012 | 6159486 | 1931 | 157.0 | 64.6 | 122.6 | 72.2 | 30.0 | 2 | 38.1 | 51.1 | 101.2 | 47.2 | 35.1 | 48.9 |
| M24S07 | 508032 | 6310048 | 1931 | 155.1 | 83.9 | 144.1 | 70.5 | 33.9 | 1 | 42.8 | 59.7 | 119.5 | 59.9 | 37.5 | 61.6 |
| CMO21 | 571137 | 6164515 | 1931 | 156.0 | 71.5 | 126.8 | 77.5 | 29.0 | 2 | 39.8 | 53.8 | 102.8 | 49.7 | 36.9 | 53.5 |
| M05S06 | 569151 | 6173167 | 1932 | 157.0 | 71.5 | 129.6 | 71.2 | 28.7 | 2 | 37.2 | 55.0 | 107.6 | 49.0 | 34.1 | 53.0 |
| B24S05 | 509485 | 6309487 | 1932 | 154.0 | 74.9 | 131.9 | 71.2 | 33.0 | 1 | 43.0 | 54.1 | 113.5 | 55.0 | 37.0 | 56.3 |
| B23S07 | 513996 | 6307007 | 1932 | 154.0 | 85.3 | 140.8 | 75.8 | 35.0 | 1 | 44.8 | 54.7 | 116.6 | 64.2 | 40.9 | 57.0 |
| 1932_12 | 595604 | 6145982 | 1932 | 152.3 | 64.9 | 115.1 | 77.8 | 31.4 | 1 | 42.0 | 64.2 | 96.7 | 52.4 | 39.2 | 63.2 |
| 1932_05 | 594996 | 6144468 | 1932 | 152.0 | 57.5 | 107.6 | 71.8 | 28.7 | 1 | 39.0 | 56.3 | 91.0 | 42.1 | 34.2 | 52.8 |
| 1932_06 | 595080 | 6144638 | 1932 | 153.0 | 67.4 | 118.4 | 75.6 | 31.7 | 2 | 41.4 | 53.6 | 103.0 | 52.7 | 39.7 | 51.6 |
| 1932_04 | 594907 | 6144243 | 1932 | 152.0 | 62.0 | 113.7 | 70.8 | 29.9 | 2 | 38.7 | 53.4 | 97.9 | 45.8 | 35.9 | 53.9 |
| 1932_07 | 595151 | 6144862 | 1932 | 151.0 | 62.7 | 115.4 | 75.2 | 30.2 | 1 | 41.6 | 55.9 | 97.5 | 49.9 | 37.6 | 57.3 |
| 1932_18 | 596493 | 6147370 | 1932 | 158.0 | 67.5 | 128.0 | 74.3 | 27.2 | 2 | 35.8 | 55.1 | 88.8 | 48.7 | 38.0 | 53.3 |
| 1932_10 | 595421 | 6145514 | 1932 | 153.9 | 74.6 | 125.4 | 81.9 | 35.9 | 1 | 49.1 | 56.2 | 108.0 | 57.8 | 44.8 | 56.9 |
| 1932_16 | 596185 | 6146968 | 1932 | 157.2 | 75.7 | 133.7 | 74.6 | 31.4 | 2 | 41.7 | 53.3 | 103.8 | 53.9 | 39.6 | 54.0 |
| 1932_09 | 595347 | 6145309 | 1932 | 151.0 | 71.9 | 121.3 | 79.4 | 34.8 | 1 | 44.8 | 60.8 | 99.3 | 54.1 | 41.5 | 58.7 |
| 1932_03 | 594803 | 6144056 | 1932 | 153.4 | 65.8 | 124.9 | 73.8 | 28.7 | 2 | 35.2 | 51.7 | 93.8 | 52.5 | 37.4 | 49.4 |
| 1932_15 | 596039 | 6146775 | 1932 | 157.0 | 70.1 | 128.2 | 75.7 | 32.0 | 2 | 39.0 | 56.3 | 104.8 | 51.0 | 37.3 | 54.4 |
| 1932_11 | 595541 | 6145817 | 1932 | 152.0 | 72.1 | 119.0 | 80.4 | 34.6 | 1 | 42.3 | 58.7 | 99.5 | 58.2 | 41.7 | 61.0 |
| 1932_08 | 595234 | 6145056 | 1932 | 154.0 | 79.8 | 134.1 | 84.1 | 35.4 | 1 | 45.5 | 59.3 | 107.4 | 62.4 | 45.9 | 58.0 |
| 1932_13 | 595795 | 6146383 | 1932 | 154.5 | 93.8 | 152.3 | 90.2 | 42.5 | 1 | 51.0 | 63.9 | 125.6 | 71.0 | 51.8 | 64.7 |
| 1932_02 | 594739 | 6143857 | 1932 | 152.0 | 71.3 | 130.0 | 75.2 | 32.1 | 2 | 39.3 | 52.8 | 112.0 | 53.5 | 38.3 | 52.1 |
| 1932_17 | 596364 | 6147175 | 1932 | 158.0 | 77.8 | 139.0 | 77.1 | 31.0 | 2 | 38.1 | 57.8 | 102.0 | 55.7 | 40.6 | 56.8 |
| 1932_14 | 595883 | 6146574 | 1932 | 156.0 | 73.6 | 130.2 | 74.2 | 32.2 | 2 | 37.9 | 54.6 | 105.0 | 53.2 | 37.6 | 55.9 |
| 1932_01 | 594773 | 6143606 | 1932 | 154.0 | 73.9 | 131.1 | 74.3 | 31.6 | 2 | 38.0 | 55.8 | 111.3 | 52.3 | 37.9 | 53.4 |
| B16S17 | 411521 | 6270017 | 1933 | 147.0 | 62.5 | 114.1 | 73.4 | 27.7 | 1 | 34.7 | 56.1 | 92.2 | 48.7 | 35.7 | 57.4 |
| M16S02 | 411648 | 6268472 | 1933 | 146.0 | 72.5 | 129.0 | 74.3 | 33.6 | 2 | 36.6 | 61.9 | 114.8 | 60.0 | 39.7 | 62.9 |
| CMO31 | 641606 | 6143766 | 1933 | 158.9 | 61.6 | 123.0 | 77.1 | 25.1 | 2 | 32.0 | 48.6 | 87.8 | 44.5 | 35.2 | 46.4 |
| CMO24 | 571789 | 6164579 | 1933 | 156.0 | 68.0 | 125.5 | 71.7 | 27.3 | 2 | 36.0 | 54.3 | 102.9 | 44.8 | 33.4 | 51.5 |
| B01S10 | 616527 | 6167014 | 1934 | 157.0 | 74.5 | 127.8 | 75.2 | 30.1 | 1 | 38.0 | 54.2 | 100.1 | 55.0 | 39.2 | 52.7 |
| B07S06 | 561263 | 6158503 | 1935 | 156.0 | 62.9 | 116.9 | 71.0 | 25.8 | 1 | 33.4 | 51.3 | 91.1 | 45.0 | 34.0 | 50.1 |
| B04S03 | 589986 | 6146112 | 1935 | 153.0 | 59.0 | 108.0 | 71.1 | 26.1 | 1 | 37.0 | 42.2 | 88.9 | 40.1 | 33.1 | 42.8 |
| B01S12 | 615993 | 6165505 | 1935 | 157.0 | 75.1 | 128.6 | 76.2 | 31.9 | 1 | 42.2 | 55.4 | 110.0 | 53.1 | 38.4 | 54.5 |
| B01S01 | 616014 | 6166992 | 1935 | 155.1 | 80.6 | 134.5 | 76.2 | 35.7 | 1 | 43.7 | 60.5 | 112.7 | 54.8 | 38.8 | 59.0 |
| B23S09 | 514510 | 6306493 | 1935 | 151.8 | 77.3 | 130.8 | 72.0 | 32.5 | 1 | 43.9 | 48.7 | 113.9 | 57.8 | 37.9 | 52.0 |
| M01S10 | 617412 | 6165285 | 1936 | 157.1 | 69.9 | 124.0 | 74.7 | 29.9 | 1 | 40.0 | 56.5 | 100.9 | 49.0 | 36.9 | 57.3 |
| M11S02 | 507817 | 6160183 | 1936 | 151.0 | 73.5 | 127.3 | 69.0 | 28.0 | 2 | 37.5 | 52.0 | 90.7 | 48.9 | 34.5 | 46.6 |
| B06S10 | 567489 | 6158977 | 1936 | 157.4 | 66.6 | 126.6 | 72.3 | 27.9 | 2 | 34.0 | 52.5 | 101.5 | 45.2 | 34.1 | 49.8 |
| M24S05 | 508997 | 6309973 | 1936 | 154.0 | 80.6 | 138.4 | 72.3 | 32.9 | 1 | 43.1 | 55.1 | 105.8 | 59.1 | 38.2 | 56.7 |
| M23S09 | 515435 | 6307641 | 1936 | 154.9 | 78.7 | 134.8 | 71.0 | 33.1 | 1 | 41.3 | 59.9 | 111.7 | 57.8 | 37.2 | 56.8 |
| CMO13 | 411441 | 6267959 | 1936 | 147.1 | 67.9 | 122.0 | 71.3 | 31.2 | 2 | 34.9 | 56.3 | 101.3 | 49.6 | 35.4 | 57.8 |
| B18S01 | 449021 | 6135475 | 1937 | 145.0 | 60.5 | 111.0 | 76.8 | 31.2 | 3 | 43.8 | 54.4 | 88.5 | 46.5 | 37.5 | 49.4 |
| B08S20 | 546007 | 6173509 | 1937 | 151.7 | 53.5 | 103.7 | 66.2 | 29.9 | 2 | 37.8 | 54.1 | 84.2 | 41.8 | 31.8 | 52.4 |
| B04S07 | 588471 | 6145487 | 1937 | 149.3 | 66.9 | 118.3 | 78.8 | 35.7 | 3 | 45.7 | 48.2 | 103.7 | 52.9 | 41.7 | 48.9 |
| M08S03 | 545769 | 6175795 | 1937 | 151.0 | 60.5 | 113.3 | 68.5 | 30.3 | 1 | 40.6 | 57.6 | 92.9 | 44.6 | 34.3 | 54.4 |
| M24S04 | 509391 | 6310134 | 1937 | 153.0 | 74.6 | 126.4 | 72.5 | 33.0 | 1 | 42.0 | 55.4 | 102.7 | 54.8 | 36.8 | 61.2 |
| B24S19 | 508516 | 6309000 | 1937 | 153.0 | 67.5 | 120.6 | 69.2 | 33.1 | 1 | 43.4 | 54.7 | 106.8 | 50.8 | 35.6 | 57.0 |
| M23S03 | 514960 | 6304442 | 1937 | 152.3 | 74.9 | 129.6 | 72.6 | 34.7 | 1 | 44.7 | 58.5 | 114.2 | 54.7 | 36.9 | 62.0 |
| B06S01 | 567525 | 6159500 | 1937 | 158.0 | 71.5 | 130.0 | 74.0 | 29.5 | 1 | 38.0 | 48.3 | 105.8 | 48.5 | 36.5 | 47.5 |
| B19S08 | 486493 | 6333001 | 1937 | 156.5 | 87.6 | 148.7 | 73.8 | 34.4 | 2 | 42.5 | 58.8 | 120.4 | 62.5 | 39.4 | 59.0 |
| CMO02 | 428020 | 6272111 | 1937 | 151.0 | 69.4 | 125.5 | 70.2 | 31.7 | 2 | 35.4 | 55.0 | 113.4 | 51.5 | 35.3 | 56.0 |
| B25S16 | 650534 | 6205252 | 1938 | 167.9 | 65.3 | 127.4 | 78.0 | 26.4 | 1 | 35.1 | 54.5 | 95.4 | 48.9 | 36.9 | 52.8 |
| B02S02 | 607475 | 6151495 | 1938 | 154.0 | 60.5 | 115.3 | 70.1 | 29.4 | 2 | 36.9 | 61.1 | 99.7 | 44.6 | 33.7 | 58.2 |
| B21S15 | 501532 | 6338510 | 1938 | 154.0 | 72.8 | 128.2 | 70.9 | 32.1 | 2 | 40.2 | 55.2 | 107.4 | 50.9 | 36.1 | 55.4 |
| B23S06 | 514404 | 6306999 | 1938 | 154.0 | 79.2 | 137.8 | 73.8 | 35.0 | 1 | 45.0 | 53.7 | 119.9 | 61.8 | 40.0 | 58.6 |
| B24S13 | 508002 | 6310563 | 1938 | 154.6 | 83.2 | 137.1 | 70.7 | 34.4 | 1 | 44.3 | 56.6 | 118.9 | 57.2 | 37.4 | 61.0 |
| M02S01 | 608199 | 6153055 | 1939 | 156.0 | 67.3 | 122.7 | 72.4 | 30.6 | 1 | 37.9 | 63.0 | 101.5 | 48.4 | 35.7 | 61.7 |
| B06S15 | 569507 | 6158508 | 1939 | 157.0 | 68.2 | 126.4 | 76.2 | 29.4 | 1 | 40.2 | 49.4 | 100.7 | 49.4 | 38.0 | 49.5 |
| B19S15 | 484160 | 6335108 | 1939 | 156.0 | 82.9 | 140.6 | 76.1 | 33.1 | 2 | 42.4 | 46.4 | 111.7 | 61.7 | 39.6 | 51.0 |
| B06S06 | 567973 | 6158510 | 1939 | 157.0 | 72.0 | 126.0 | 72.0 | 28.7 | 1 | 37.3 | 47.9 | 102.5 | 46.0 | 35.0 | 47.4 |
| B01S20 | 616999 | 6165502 | 1940 | 155.0 | 64.7 | 116.8 | 72.9 | 29.2 | 1 | 35.8 | 52.7 | 93.0 | 45.8 | 35.7 | 51.6 |
| M05S03 | 569148 | 6174905 | 1940 | 156.0 | 68.5 | 125.0 | 73.2 | 30.1 | 2 | 37.5 | 53.2 | 103.2 | 49.1 | 35.8 | 51.6 |
| M04S07 | 589183 | 6146980 | 1940 | 151.9 | 67.7 | 122.7 | 78.7 | 32.8 | 1 | 40.8 | 49.3 | 101.7 | 53.1 | 39.8 | 49.9 |
| B16S10 | 411991 | 6268025 | 1940 | 148.0 | 75.7 | 134.6 | 75.2 | 31.9 | 2 | 37.4 | 68.7 | 107.3 | 59.1 | 39.8 | 65.6 |
| B16S03 | 410514 | 6268007 | 1940 | 148.8 | 73.2 | 130.1 | 74.0 | 32.8 | 2 | 39.0 | 59.1 | 114.4 | 54.9 | 37.9 | 60.0 |
| B02S01 | 607467 | 6151981 | 1940 | 154.2 | 84.2 | 143.3 | 81.9 | 37.3 | 1 | 45.6 | 62.3 | 118.6 | 63.1 | 45.0 | 65.1 |
| B24S18 | 508000 | 6309000 | 1940 | 152.5 | 84.0 | 143.0 | 75.3 | 37.3 | 1 | 45.5 | 62.2 | 120.0 | 61.3 | 40.5 | 60.6 |
| B07S10 | 560777 | 6157983 | 1941 | 156.0 | 61.2 | 114.5 | 70.2 | 27.0 | 1 | 34.4 | 50.3 | 93.3 | 43.5 | 34.8 | 48.3 |
| B21S12 | 502014 | 6338574 | 1941 | 153.5 | 71.1 | 125.3 | 71.0 | 32.0 | 1 | 42.6 | 57.9 | 105.5 | 53.5 | 36.9 | 61.6 |
| M15S08 | 426970 | 6268003 | 1941 | 149.4 | 62.9 | 113.3 | 68.5 | 28.2 | 2 | 34.0 | 56.0 | 100.4 | 46.2 | 35.1 | 57.4 |
| B26S03 | 647330 | 6209439 | 1941 | 161.0 | 58.2 | 109.2 | 66.1 | 28.3 | 2 | 34.3 | 51.3 | 99.7 | 40.1 | 31.0 | 51.1 |
| M02S04 | 609938 | 6152022 | 1941 | 158.0 | 78.5 | 136.5 | 77.5 | 33.4 | 1 | 42.0 | 57.0 | 108.0 | 56.0 | 38.9 | 59.5 |
| M01S08 | 615695 | 6165210 | 1941 | 156.0 | 79.0 | 136.3 | 80.1 | 34.6 | 1 | 41.8 | 62.1 | 111.1 | 59.5 | 40.2 | 63.6 |
| B24S15 | 507976 | 6309489 | 1941 | 153.0 | 86.1 | 142.9 | 76.3 | 37.0 | 1 | 45.6 | 59.5 | 122.4 | 67.7 | 41.9 | 57.5 |
| B24S02 | 509997 | 6308501 | 1941 | 155.0 | 83.1 | 143.0 | 70.6 | 36.1 | 1 | 46.0 | 59.2 | 127.9 | 58.4 | 38.3 | 56.4 |
| CMO27 | 572341 | 6164654 | 1941 | 155.0 | 72.0 | 127.3 | 71.1 | 28.8 | 2 | 36.4 | 59.2 | 107.0 | 51.4 | 35.9 | 58.0 |
| B02S15 | 609524 | 6150551 | 1942 | 155.0 | 65.0 | 114.0 | 74.0 | 30.0 | 1 | 40.8 | 53.4 | 91.4 | 48.0 | 37.0 | 53.9 |
| M06S05 | 568861 | 6158982 | 1942 | 159.0 | 72.9 | 131.9 | 75.3 | 30.0 | 2 | 39.4 | 51.5 | 103.4 | 51.3 | 36.9 | 49.5 |
| B02S12 | 609478 | 6152000 | 1942 | 158.0 | 76.3 | 132.9 | 79.4 | 28.7 | 1 | 39.0 | 58.1 | 102.9 | 57.3 | 40.3 | 55.8 |
| B07S17 | 560727 | 6157503 | 1943 | 156.3 | 72.9 | 127.1 | 75.8 | 29.6 | 1 | 38.1 | 49.5 | 100.6 | 50.8 | 37.4 | 51.6 |
| B06S20 | 569017 | 6158486 | 1943 | 158.0 | 78.7 | 137.3 | 79.8 | 30.7 | 1 | 41.0 | 51.0 | 103.4 | 60.3 | 42.7 | 50.0 |
| M02S08 | 606997 | 6151033 | 1944 | 155.0 | 63.7 | 115.3 | 71.7 | 29.6 | 1 | 38.8 | 62.1 | 95.4 | 47.5 | 35.8 | 58.8 |
| B07S05 | 560737 | 6158518 | 1944 | 155.0 | 62.0 | 113.2 | 71.0 | 26.2 | 2 | 34.2 | 50.2 | 92.4 | 45.2 | 33.2 | 49.6 |
| B23S11 | 514765 | 6303929 | 1944 | 151.0 | 65.7 | 117.6 | 68.4 | 31.6 | 1 | 40.8 | 49.8 | 100.3 | 48.1 | 34.7 | 53.1 |
| B16S12 | 412477 | 6269494 | 1944 | 149.0 | 72.0 | 128.6 | 72.5 | 35.0 | 2 | 38.2 | 60.6 | 118.0 | 56.9 | 38.4 | 62.5 |
| B02S17 | 609007 | 6151029 | 1944 | 158.0 | 88.1 | 149.4 | 78.7 | 34.4 | 1 | 44.5 | 61.9 | 121.3 | 59.8 | 42.5 | 59.5 |
| M18S04 | 448986 | 6135617 | 1945 | 146.0 | 67.5 | 122.1 | 75.8 | 41.4 | 3 | 54.7 | 61.6 | 117.1 | 45.8 | 37.8 | 57.3 |
| M08S02 | 545331 | 6175875 | 1945 | 153.0 | 58.4 | 109.5 | 67.9 | 29.7 | 2 | 40.2 | 54.9 | 89.7 | 43.4 | 33.3 | 53.6 |
| M03S07 | 589341 | 6153737 | 1945 | 152.9 | 66.5 | 121.0 | 75.8 | 29.7 | 1 | 39.8 | 50.7 | 95.6 | 50.9 | 38.6 | 51.7 |
| B07S19 | 561776 | 6157497 | 1945 | 157.0 | 71.7 | 125.9 | 73.5 | 30.0 | 2 | 38.7 | 54.6 | 102.5 | 51.8 | 37.5 | 55.7 |
| B06S18 | 569028 | 6157509 | 1945 | 157.9 | 74.4 | 130.6 | 76.9 | 32.2 | 1 | 41.5 | 55.0 | 107.5 | 51.7 | 38.0 | 55.3 |
| B15S13 | 428133 | 6267151 | 1945 | 150.0 | 66.8 | 120.2 | 70.8 | 32.1 | 2 | 36.3 | 60.2 | 107.4 | 48.8 | 34.0 | 57.4 |
| M04S10 | 588702 | 6145604 | 1946 | 151.6 | 62.8 | 118.2 | 76.8 | 29.9 | 2 | 39.6 | 50.7 | 93.2 | 49.5 | 37.3 | 48.3 |
| M16S10 | 412075 | 6270731 | 1946 | 148.0 | 68.8 | 123.3 | 72.1 | 31.7 | 2 | 37.5 | 62.5 | 109.1 | 49.1 | 35.6 | 62.5 |
| B23S08 | 514000 | 6306501 | 1946 | 152.0 | 70.4 | 125.5 | 70.5 | 33.2 | 1 | 41.7 | 52.9 | 108.3 | 51.7 | 36.2 | 54.2 |
| B16S06 | 411510 | 6268507 | 1946 | 147.0 | 67.2 | 119.1 | 69.7 | 31.7 | 2 | 33.4 | 63.2 | 103.9 | 48.5 | 35.5 | 61.4 |
| B16S08 | 412504 | 6268479 | 1947 | 148.0 | 75.9 | 132.0 | 73.6 | 32.6 | 2 | 37.6 | 65.8 | 113.4 | 56.5 | 37.7 | 64.0 |
| B01S18 | 617024 | 6164499 | 1947 | 158.0 | 89.0 | 152.0 | 80.0 | 35.5 | 1 | 43.8 | 59.5 | 118.0 | 60.0 | 43.0 | 57.3 |
| CMM16 | 411671 | 6266864 | 1947 | 149.0 | 65.9 | 120.3 | 69.2 | 26.8 | 2 | 34.0 | 56.8 | 105.6 | 47.8 | 35.3 | 56.4 |
| M02S02 | 609471 | 6152362 | 1948 | 156.0 | 74.0 | 132.0 | 74.7 | 29.3 | 1 | 39.3 | 60.2 | 99.6 | 51.3 | 37.3 | 61.2 |
| B08S07 | 545503 | 6175523 | 1948 | 152.2 | 55.1 | 104.7 | 64.2 | 28.4 | 2 | 38.7 | 53.3 | 92.2 | 38.8 | 30.1 | 51.0 |
| M17S01 | 451720 | 6127947 | 1948 | 144.0 | 50.3 | 96.4 | 72.5 | 38.0 | 3 | 53.7 | 80.1 | 109.5 | 40.6 | 35.4 | 72.8 |
| B07S03 | 559732 | 6158503 | 1948 | 154.0 | 55.0 | 108.0 | 72.0 | 28.3 | 1 | 36.3 | 51.0 | 91.2 | 43.0 | 33.0 | 50.8 |
| B05S09 | 569471 | 6175508 | 1948 | 155.0 | 59.2 | 115.2 | 71.4 | 27.5 | 2 | 35.1 | 52.8 | 95.2 | 43.7 | 34.2 | 50.2 |
| B05S08 | 569506 | 6176010 | 1948 | 156.0 | 62.1 | 118.9 | 72.6 | 27.7 | 2 | 36.9 | 51.6 | 99.9 | 45.9 | 34.2 | 50.7 |
| B16S09 | 412474 | 6268007 | 1948 | 147.0 | 67.8 | 125.2 | 72.3 | 31.1 | 2 | 36.3 | 56.3 | 107.8 | 50.9 | 36.1 | 59.4 |
| M26S02 | 647094 | 6211173 | 1948 | 161.0 | 64.5 | 115.2 | 67.3 | 26.6 | 2 | 34.4 | 59.6 | 103.1 | 42.3 | 31.7 | 56.0 |
| B16S11 | 411996 | 6269491 | 1948 | 148.0 | 75.4 | 130.9 | 77.4 | 32.8 | 2 | 37.0 | 60.0 | 112.7 | 63.6 | 40.3 | 60.3 |
| B24S06 | 509489 | 6310010 | 1949 | 153.0 | 68.0 | 123.8 | 68.5 | 34.1 | 1 | 44.9 | 64.9 | 110.5 | 47.3 | 34.2 | 64.0 |
| B06S19 | 568974 | 6157989 | 1949 | 157.5 | 70.4 | 130.2 | 76.0 | 29.2 | 2 | 38.3 | 55.3 | 104.0 | 49.9 | 37.2 | 52.4 |
| B01S07 | 617518 | 6166497 | 1949 | 156.0 | 70.1 | 124.3 | 75.1 | 31.5 | 1 | 39.8 | 56.4 | 101.8 | 52.4 | 38.6 | 56.7 |
| M06S06 | 568024 | 6159238 | 1949 | 157.5 | 66.7 | 119.5 | 69.4 | 29.0 | 2 | 36.0 | 51.0 | 102.5 | 48.8 | 35.0 | 49.6 |
| M06S10 | 569309 | 6158807 | 1949 | 157.0 | 73.1 | 131.7 | 76.3 | 32.4 | 1 | 41.0 | 53.5 | 105.1 | 51.4 | 39.1 | 54.2 |
| B06S07 | 567977 | 6158014 | 1949 | 157.0 | 73.9 | 132.8 | 76.8 | 30.4 | 1 | 40.5 | 51.3 | 105.1 | 54.8 | 38.9 | 50.8 |
| B24S07 | 510000 | 6309993 | 1949 | 155.2 | 80.3 | 137.8 | 73.9 | 35.0 | 2 | 43.7 | 63.1 | 120.5 | 61.4 | 39.6 | 59.4 |
| M02S07 | 606461 | 6151427 | 1950 | 153.0 | 73.3 | 130.7 | 70.4 | 29.2 | 1 | 38.6 | 65.7 | 101.5 | 50.7 | 36.6 | 62.9 |
| M07S03 | 560894 | 6158353 | 1950 | 154.0 | 62.3 | 113.8 | 74.7 | 27.2 | 1 | 35.6 | 49.6 | 92.8 | 47.0 | 35.0 | 48.4 |
| B04S09 | 587993 | 6146018 | 1950 | 149.2 | 49.8 | 95.7 | 70.9 | 25.2 | 3 | 35.0 | 44.6 | 77.1 | 38.4 | 31.1 | 45.4 |
| M06S03 | 567043 | 6158239 | 1950 | 156.1 | 72.4 | 129.6 | 77.6 | 31.5 | 1 | 40.3 | 51.7 | 105.1 | 53.3 | 38.6 | 50.0 |
| B15S10 | 426514 | 6264883 | 1950 | 151.0 | 63.3 | 118.3 | 73.0 | 31.9 | 2 | 34.7 | 58.7 | 109.9 | 49.0 | 35.4 | 58.0 |
| M16S08 | 411905 | 6270423 | 1951 | 148.8 | 66.3 | 118.3 | 73.0 | 30.6 | 1 | 34.5 | 58.9 | 99.8 | 50.8 | 36.0 | 60.4 |
| M07S02 | 560399 | 6158177 | 1951 | 157.0 | 60.3 | 114.1 | 71.2 | 26.1 | 2 | 34.9 | 48.5 | 92.5 | 42.5 | 33.9 | 48.2 |
| B02S10 | 608011 | 6152020 | 1951 | 154.0 | 67.2 | 119.5 | 71.0 | 31.3 | 2 | 39.8 | 64.3 | 102.5 | 47.7 | 35.1 | 62.5 |
| B16S18 | 411027 | 6270026 | 1951 | 149.0 | 62.4 | 115.3 | 72.6 | 30.0 | 1 | 36.0 | 51.6 | 98.1 | 48.3 | 34.9 | 52.7 |
| M01S05 | 616401 | 6166147 | 1951 | 157.0 | 72.5 | 126.7 | 77.0 | 31.5 | 1 | 39.6 | 53.1 | 104.0 | 52.0 | 38.5 | 55.0 |
| M06S08 | 569121 | 6158361 | 1951 | 157.3 | 76.3 | 132.0 | 77.7 | 32.4 | 1 | 41.1 | 50.1 | 110.2 | 55.3 | 39.3 | 50.1 |
| 1951_5 | 619183 | 6161082 | 1951 | 156.1 | 67.6 | 121.3 | 71.0 | 29.6 | 1 | 38.5 | 56.3 | 102.0 | 47.0 | 35.9 | 56.5 |
| 1951_7 | 619313 | 6161136 | 1951 | 155.0 | 64.3 | 118.2 | 69.1 | 28.1 | 1 | 37.2 | 52.6 | 99.1 | 45.2 | 34.2 | 54.5 |
| 1951_8 | 618998 | 6161062 | 1951 | 155.0 | 65.0 | 116.8 | 72.2 | 29.6 | 1 | 37.8 | 52.1 | 94.8 | 49.3 | 35.7 | 51.1 |
| 1951_4 | 618802 | 6161311 | 1951 | 154.0 | 66.3 | 120.8 | 73.3 | 28.4 | 1 | 37.8 | 49.0 | 93.7 | 49.6 | 36.3 | 50.6 |
| 1951_2 | 618649 | 6161492 | 1951 | 156.0 | 62.9 | 114.0 | 70.1 | 27.5 | 1 | 35.9 | 48.2 | 95.1 | 43.8 | 34.6 | 48.0 |
| 1951_3 | 618843 | 6161493 | 1951 | 155.2 | 68.4 | 121.7 | 72.2 | 29.2 | 1 | 40.0 | 51.6 | 100.7 | 49.7 | 36.4 | 50.1 |
| 1951_1 | 618652 | 6161287 | 1951 | 155.0 | 67.2 | 120.0 | 72.3 | 30.4 | 1 | 37.0 | 50.0 | 100.4 | 45.6 | 35.3 | 51.0 |
| 1951_6 | 619238 | 6161288 | 1951 | 156.0 | 67.0 | 123.8 | 73.0 | 30.0 | 1 | 38.0 | 54.6 | 102.0 | 49.9 | 36.0 | 52.8 |
| B08S06 | 545014 | 6175523 | 1952 | 151.4 | 50.2 | 101.8 | 62.1 | 27.6 | 2 | 36.5 | 54.8 | 85.3 | 38.0 | 28.0 | 52.1 |
| B14S17 | 426015 | 6258983 | 1952 | 149.0 | 65.5 | 115.4 | 73.9 | 28.2 | 2 | 36.0 | 53.9 | 98.0 | 47.4 | 36.0 | 52.1 |
| B09S19 | 546036 | 6166513 | 1952 | 156.0 | 70.9 | 121.3 | 76.9 | 35.0 | 1 | 43.0 | 57.6 | 103.0 | 52.9 | 38.0 | 58.3 |
| B06S09 | 567480 | 6158497 | 1952 | 156.3 | 62.7 | 117.8 | 68.3 | 27.9 | 2 | 34.8 | 49.2 | 98.5 | 44.3 | 33.7 | 48.8 |
| M23S06 | 515268 | 6306299 | 1952 | 150.6 | 75.9 | 128.0 | 71.1 | 33.4 | 1 | 42.9 | 59.1 | 110.3 | 54.8 | 36.2 | 62.5 |
| M23S02 | 514971 | 6304028 | 1952 | 151.0 | 72.3 | 124.3 | 68.5 | 31.9 | 1 | 41.9 | 55.5 | 104.2 | 53.0 | 35.4 | 57.1 |
| B01S16 | 615997 | 6164505 | 1952 | 157.0 | 81.6 | 140.1 | 80.2 | 35.2 | 1 | 43.2 | 54.6 | 113.7 | 59.9 | 41.9 | 52.4 |
| M26S03 | 646964 | 6210924 | 1952 | 163.0 | 70.3 | 123.8 | 69.4 | 28.4 | 2 | 35.6 | 62.1 | 102.3 | 46.8 | 34.8 | 60.3 |
| B01S14 | 615510 | 6164999 | 1952 | 159.5 | 85.3 | 145.8 | 77.5 | 34.9 | 1 | 41.8 | 57.0 | 118.5 | 59.8 | 40.9 | 55.8 |
| CMM17 | 411666 | 6265998 | 1952 | 150.0 | 62.7 | 114.7 | 69.7 | 26.6 | 2 | 32.4 | 54.1 | 97.9 | 47.5 | 32.9 | 53.7 |
| B11S02 | 507983 | 6159521 | 1953 | 149.9 | 57.7 | 109.4 | 71.9 | 28.5 | 1 | 38.9 | 53.3 | 81.6 | 42.0 | 34.2 | 50.7 |
| B03S11 | 592022 | 6155004 | 1953 | 152.6 | 66.7 | 120.5 | 78.6 | 29.6 | 1 | 40.4 | 53.7 | 92.5 | 49.3 | 38.9 | 52.9 |
| B06S11 | 569008 | 6158965 | 1953 | 158.2 | 72.3 | 128.6 | 75.8 | 31.9 | 2 | 40.3 | 49.2 | 107.7 | 50.9 | 38.1 | 47.0 |
| B21S05 | 503508 | 6338477 | 1953 | 157.0 | 78.3 | 135.4 | 72.3 | 33.4 | 2 | 43.0 | 55.1 | 113.6 | 55.3 | 37.2 | 59.6 |
| B16S05 | 410977 | 6268512 | 1953 | 147.0 | 63.8 | 118.9 | 70.6 | 29.2 | 2 | 34.6 | 64.5 | 101.5 | 51.0 | 35.0 | 64.0 |
| M24S06 | 508518 | 6310057 | 1953 | 153.0 | 74.5 | 130.1 | 73.5 | 32.6 | 1 | 42.3 | 51.2 | 104.4 | 55.2 | 37.0 | 55.5 |
| M14S09 | 427154 | 6257980 | 1954 | 152.9 | 70.5 | 123.3 | 74.3 | 28.8 | 2 | 36.4 | 39.0 | 99.3 | 50.1 | 36.3 | 42.8 |
| B14S18 | 425987 | 6258510 | 1954 | 149.0 | 68.6 | 122.6 | 73.1 | 31.9 | 2 | 39.9 | 50.8 | 110.1 | 50.0 | 36.2 | 51.5 |
| B07S07 | 561766 | 6158478 | 1954 | 156.0 | 67.5 | 119.6 | 75.9 | 30.4 | 1 | 39.0 | 51.9 | 97.9 | 50.6 | 37.7 | 51.7 |
| M03S05 | 589905 | 6154175 | 1954 | 154.0 | 74.1 | 129.3 | 78.5 | 31.9 | 1 | 41.9 | 57.1 | 101.7 | 53.0 | 40.0 | 56.7 |
| M01S09 | 617217 | 6165168 | 1954 | 157.8 | 74.3 | 129.0 | 76.4 | 33.4 | 1 | 43.0 | 55.1 | 105.3 | 52.1 | 38.5 | 56.4 |
| B07S15 | 560220 | 6157006 | 1954 | 155.0 | 67.8 | 123.6 | 69.6 | 28.2 | 2 | 34.6 | 53.2 | 97.0 | 47.8 | 34.4 | 54.2 |
| B01S17 | 616523 | 6164495 | 1954 | 156.0 | 84.0 | 141.6 | 81.8 | 36.7 | 1 | 43.6 | 61.6 | 114.0 | 61.3 | 43.8 | 60.5 |
| B23S04 | 515549 | 6307076 | 1954 | 155.0 | 86.9 | 145.1 | 74.5 | 36.3 | 1 | 44.8 | 58.2 | 123.9 | 65.0 | 41.1 | 59.2 |
| M23S04 | 514920 | 6304966 | 1955 | 150.4 | 70.8 | 122.5 | 69.3 | 32.0 | 1 | 42.9 | 62.1 | 104.4 | 49.9 | 35.4 | 65.0 |
| B03S16 | 591996 | 6153524 | 1955 | 152.5 | 66.7 | 117.3 | 75.8 | 28.9 | 1 | 39.5 | 53.0 | 92.9 | 47.1 | 36.3 | 49.2 |
| B03S17 | 591510 | 6153474 | 1955 | 154.0 | 64.8 | 119.2 | 75.3 | 29.6 | 1 | 38.1 | 52.4 | 93.5 | 49.2 | 37.7 | 52.1 |
| B06S05 | 568008 | 6159011 | 1955 | 157.0 | 67.4 | 125.5 | 71.5 | 28.6 | 2 | 37.0 | 50.6 | 100.2 | 45.3 | 34.1 | 49.6 |
| B21S04 | 503960 | 6338538 | 1955 | 155.0 | 68.2 | 123.5 | 68.3 | 30.9 | 2 | 38.9 | 59.0 | 101.0 | 48.8 | 34.3 | 58.2 |
| B01S04 | 616017 | 6166497 | 1955 | 156.0 | 71.7 | 124.6 | 72.0 | 31.6 | 1 | 39.4 | 55.2 | 104.1 | 51.3 | 37.1 | 54.0 |
| B17S14 | 451977 | 6127977 | 1956 | 145.6 | 52.3 | 98.5 | 69.8 | 24.0 | 3 | 35.8 | 53.6 | 69.9 | 37.6 | 32.8 | 45.4 |
| M15S05 | 426921 | 6265637 | 1956 | 151.3 | 77.3 | 134.6 | 73.7 | 32.3 | 1 | 40.3 | 63.7 | 107.3 | 56.3 | 39.7 | 64.0 |
| M01S03 | 616172 | 6166705 | 1956 | 157.6 | 76.9 | 132.6 | 78.2 | 33.0 | 1 | 42.3 | 55.4 | 106.3 | 56.1 | 40.2 | 55.0 |
| B15S01 | 427011 | 6264920 | 1956 | 151.0 | 76.9 | 138.6 | 77.3 | 32.0 | 2 | 38.6 | 63.2 | 106.8 | 58.4 | 38.7 | 63.5 |
| M01S06 | 616629 | 6165682 | 1956 | 157.1 | 81.2 | 138.6 | 77.8 | 33.0 | 1 | 43.0 | 58.5 | 113.4 | 59.5 | 42.8 | 57.7 |
| M08S01 | 545574 | 6175892 | 1957 | 154.0 | 63.0 | 120.7 | 69.6 | 34.0 | 2 | 43.0 | 59.3 | 102.4 | 48.6 | 35.8 | 56.5 |
| B07S18 | 561265 | 6157491 | 1957 | 155.0 | 57.3 | 109.4 | 68.2 | 26.0 | 1 | 32.2 | 49.2 | 88.5 | 38.7 | 30.3 | 48.7 |
| B21S03 | 504348 | 6338618 | 1957 | 154.5 | 71.1 | 127.1 | 73.7 | 33.9 | 2 | 41.4 | 62.6 | 110.4 | 52.8 | 36.9 | 63.9 |
| B06S16 | 569496 | 6158010 | 1957 | 158.0 | 71.7 | 130.4 | 75.3 | 30.5 | 1 | 39.9 | 49.3 | 101.4 | 50.7 | 38.1 | 49.1 |
| B16S04 | 410531 | 6268488 | 1957 | 146.0 | 65.9 | 121.7 | 71.3 | 32.0 | 2 | 35.7 | 58.5 | 107.3 | 53.4 | 38.4 | 58.5 |
| 1957_12 | 545766 | 6165879 | 1957 | 156.0 | 62.0 | 113.3 | 73.0 | 31.1 | 1 | 39.0 | 59.7 | 86.4 | 47.0 | 35.0 | 62.3 |
| 1957_04 | 545711 | 6163964 | 1957 | 154.0 | 58.4 | 108.5 | 71.5 | 28.9 | 1 | 36.9 | 57.1 | 84.7 | 44.5 | 33.8 | 54.4 |
| 1957_05 | 545881 | 6164692 | 1957 | 155.0 | 55.9 | 105.6 | 71.5 | 28.8 | 1 | 39.9 | 55.5 | 79.4 | 43.1 | 33.6 | 54.5 |
| 1957_13 | 545275 | 6162707 | 1957 | 155.0 | 60.0 | 109.0 | 71.5 | 30.8 | 2 | 39.9 | 53.2 | 93.4 | 44.5 | 34.0 | 51.9 |
| 1957_09 | 545819 | 6165230 | 1957 | 155.5 | 57.4 | 108.7 | 69.0 | 27.0 | 1 | 36.3 | 51.2 | 85.0 | 40.6 | 31.8 | 52.5 |
| 1957_14 | 545463 | 6163284 | 1957 | 155.0 | 56.7 | 109.6 | 68.1 | 26.1 | 1 | 35.3 | 53.4 | 83.3 | 40.3 | 31.0 | 52.6 |
| 1957_06 | 545873 | 6164513 | 1957 | 155.0 | 55.3 | 104.2 | 69.6 | 29.4 | 1 | 38.0 | 53.4 | 86.9 | 41.3 | 33.0 | 53.0 |
| 1957_03 | 545734 | 6164151 | 1957 | 154.0 | 65.3 | 117.4 | 76.7 | 29.5 | 1 | 42.3 | 52.3 | 94.9 | 47.1 | 36.9 | 52.1 |
| 1957_02 | 545530 | 6163559 | 1957 | 155.3 | 56.0 | 106.4 | 69.6 | 26.3 | 1 | 35.8 | 49.2 | 83.2 | 41.6 | 32.3 | 52.0 |
| 1957_07 | 545835 | 6165094 | 1957 | 154.0 | 58.5 | 104.4 | 70.0 | 26.6 | 1 | 37.8 | 49.9 | 78.4 | 43.5 | 33.8 | 49.8 |
| 1957_10 | 545809 | 6165403 | 1957 | 155.0 | 51.9 | 101.3 | 70.7 | 28.9 | 1 | 38.4 | 53.1 | 81.8 | 39.1 | 31.0 | 50.8 |
| 1957_01 | 545608 | 6163777 | 1957 | 155.5 | 53.0 | 103.4 | 68.7 | 25.6 | 1 | 36.7 | 50.2 | 78.8 | 40.1 | 31.4 | 49.3 |
| 1957_11 | 545789 | 6165665 | 1957 | 156.0 | 68.0 | 121.0 | 75.8 | 35.9 | 1 | 43.7 | 63.1 | 108.2 | 53.3 | 38.9 | 66.0 |
| 1957_08 | 545875 | 6164906 | 1957 | 155.0 | 70.7 | 124.0 | 73.0 | 32.4 | 1 | 42.7 | 52.7 | 102.2 | 52.6 | 38.0 | 52.0 |
| B17S13 | 451478 | 6127986 | 1958 | 145.0 | 54.2 | 100.9 | 73.4 | 25.8 | 3 | 38.1 | 56.3 | 75.9 | 37.5 | 32.5 | 48.6 |
| B17S02 | 452525 | 6125896 | 1958 | 146.0 | 44.7 | 91.4 | 66.7 | 26.0 | 3 | 36.2 | 52.1 | 77.1 | 32.8 | 30.0 | 43.8 |
| B03S15 | 592499 | 6153528 | 1958 | 154.2 | 68.5 | 121.1 | 77.7 | 32.3 | 1 | 42.8 | 50.0 | 102.9 | 50.0 | 38.5 | 50.2 |
| B06S14 | 569494 | 6158982 | 1958 | 158.0 | 68.2 | 125.6 | 73.5 | 27.8 | 2 | 36.9 | 48.4 | 103.4 | 48.1 | 35.7 | 48.4 |
| B07S08 | 561740 | 6158005 | 1958 | 155.1 | 68.6 | 123.9 | 73.4 | 31.3 | 1 | 38.3 | 55.3 | 102.0 | 49.4 | 37.0 | 54.9 |
| B01S11 | 616470 | 6165503 | 1958 | 157.2 | 79.3 | 134.8 | 79.3 | 34.5 | 1 | 43.6 | 55.1 | 109.1 | 56.1 | 40.2 | 55.9 |
| B15S09 | 425998 | 6264907 | 1958 | 151.0 | 68.1 | 124.9 | 73.3 | 29.8 | 2 | 33.5 | 63.4 | 104.3 | 52.0 | 37.2 | 61.2 |
| M13S11 | 434667 | 6265526 | 1959 | 151.5 | 72.1 | 127.3 | 72.6 | 31.0 | 2 | 42.4 | 55.6 | 116.3 | 53.5 | 35.4 | 54.8 |
| B15S03 | 427992 | 6264893 | 1959 | 150.0 | 70.9 | 126.8 | 73.9 | 30.4 | 2 | 36.4 | 55.5 | 101.7 | 52.8 | 37.9 | 60.9 |
| B16S01 | 411509 | 6268007 | 1959 | 147.9 | 65.4 | 119.2 | 70.8 | 29.8 | 2 | 34.4 | 59.0 | 103.7 | 50.8 | 35.4 | 59.1 |
| B25S08 | 651999 | 6204502 | 1959 | 171.0 | 77.5 | 142.4 | 78.4 | 27.9 | 2 | 34.7 | 54.5 | 101.0 | 53.0 | 37.9 | 52.5 |
| B06S17 | 569521 | 6157503 | 1960 | 157.0 | 69.9 | 126.9 | 75.5 | 28.8 | 1 | 36.9 | 51.3 | 100.5 | 49.3 | 37.1 | 53.2 |
| B07S09 | 561242 | 6158003 | 1960 | 155.1 | 69.3 | 123.2 | 74.8 | 30.9 | 1 | 38.9 | 54.4 | 100.4 | 50.5 | 37.1 | 53.4 |
| B02S18 | 609000 | 6151537 | 1960 | 156.5 | 74.0 | 128.4 | 76.0 | 30.5 | 1 | 40.5 | 53.0 | 100.0 | 53.5 | 38.0 | 54.0 |
| M18S05 | 448862 | 6135306 | 1961 | 147.3 | 69.1 | 122.3 | 79.3 | 33.7 | 3 | 46.3 | 79.8 | 102.8 | 53.6 | 41.8 | 78.5 |
| M14S13 | 426999 | 6258936 | 1961 | 150.5 | 68.0 | 120.2 | 75.5 | 30.5 | 2 | 38.5 | 51.9 | 101.1 | 52.4 | 37.5 | 53.2 |
| M15S01 | 426920 | 6265053 | 1961 | 153.0 | 68.5 | 126.7 | 77.2 | 30.9 | 1 | 37.7 | 60.1 | 105.2 | 51.7 | 37.4 | 61.9 |
| B15S15 | 427517 | 6267445 | 1961 | 148.7 | 65.5 | 121.1 | 73.8 | 29.9 | 2 | 35.8 | 60.6 | 101.0 | 50.0 | 36.7 | 55.9 |
| M03S04 | 590124 | 6154539 | 1961 | 153.0 | 70.1 | 124.2 | 75.7 | 29.6 | 1 | 37.6 | 51.4 | 96.7 | 50.1 | 37.7 | 52.3 |
| B14S13 | 426504 | 6258500 | 1961 | 150.5 | 73.3 | 130.9 | 74.5 | 31.0 | 2 | 36.7 | 51.8 | 105.8 | 52.2 | 37.8 | 51.6 |
| M15S09 | 426908 | 6268279 | 1961 | 149.0 | 64.4 | 117.9 | 70.2 | 28.4 | 2 | 33.5 | 53.6 | 102.8 | 45.5 | 33.5 | 56.2 |
| M23S10 | 515193 | 6307726 | 1961 | 153.1 | 79.1 | 135.8 | 72.5 | 34.3 | 1 | 45.2 | 58.1 | 115.6 | 60.5 | 40.3 | 63.4 |
| M26S10 | 646442 | 6208183 | 1961 | 163.0 | 71.4 | 127.4 | 68.5 | 29.8 | 2 | 35.0 | 54.7 | 112.7 | 50.6 | 34.0 | 51.9 |
| B01S15 | 615500 | 6164524 | 1962 | 155.5 | 67.2 | 122.7 | 73.8 | 29.8 | 1 | 38.6 | 59.0 | 100.8 | 48.4 | 36.6 | 57.1 |
| B05S10 | 569517 | 6174988 | 1962 | 155.0 | 57.6 | 111.2 | 67.2 | 25.2 | 1 | 36.4 | 51.7 | 91.5 | 40.1 | 30.8 | 50.0 |
| B14S14 | 427000 | 6258500 | 1962 | 149.0 | 67.5 | 121.0 | 73.3 | 29.8 | 2 | 35.8 | 48.5 | 99.0 | 50.3 | 36.0 | 53.7 |
| B10S01 | 544768 | 6156993 | 1963 | 156.0 | 65.4 | 117.1 | 74.7 | 33.4 | 1 | 44.3 | 66.0 | 101.5 | 48.7 | 38.2 | 64.4 |
| B16S19 | 411014 | 6269408 | 1963 | 148.0 | 62.6 | 113.4 | 70.1 | 27.9 | 1 | 34.2 | 50.7 | 93.1 | 47.0 | 33.2 | 55.3 |
| B24S10 | 510485 | 6308985 | 1963 | 154.0 | 79.0 | 134.9 | 71.9 | 34.7 | 1 | 44.0 | 67.0 | 120.6 | 58.3 | 38.9 | 67.1 |
| B02S19 | 608977 | 6152007 | 1963 | 156.9 | 80.8 | 140.7 | 80.5 | 32.3 | 1 | 42.9 | 61.0 | 105.2 | 58.9 | 42.5 | 58.5 |
| B26S02 | 647249 | 6208954 | 1963 | 162.3 | 73.4 | 129.3 | 67.9 | 28.6 | 2 | 35.3 | 56.5 | 107.9 | 48.4 | 34.2 | 55.8 |
| B10S16 | 541282 | 6157310 | 1964 | 151.3 | 56.7 | 105.1 | 65.2 | 31.3 | 1 | 44.2 | 60.8 | 92.2 | 39.9 | 30.3 | 59.4 |
| B13S16 | 437996 | 6265502 | 1964 | 149.4 | 61.0 | 114.5 | 69.4 | 29.9 | 2 | 40.9 | 54.6 | 104.2 | 46.7 | 33.9 | 53.3 |
| B13S17 | 438004 | 6265015 | 1964 | 150.0 | 66.1 | 122.6 | 74.9 | 32.3 | 2 | 42.8 | 50.1 | 110.8 | 53.7 | 36.9 | 52.5 |
| M11S04 | 505753 | 6159618 | 1964 | 146.2 | 63.5 | 117.1 | 65.4 | 29.4 | 2 | 40.5 | 59.5 | 105.6 | 45.8 | 32.3 | 55.4 |
| M16S03 | 411548 | 6268739 | 1964 | 147.0 | 61.9 | 114.7 | 73.0 | 29.6 | 2 | 34.4 | 57.9 | 96.4 | 48.6 | 35.6 | 58.0 |
| B15S20 | 426488 | 6266997 | 1964 | 149.0 | 59.9 | 110.1 | 70.0 | 28.6 | 2 | 34.0 | 49.9 | 95.3 | 45.3 | 33.6 | 54.3 |
| M16S05 | 411634 | 6269301 | 1964 | 149.0 | 66.1 | 119.6 | 74.3 | 29.9 | 2 | 35.5 | 55.6 | 99.0 | 53.5 | 36.8 | 59.1 |
| 1964_3 | 574426 | 6154209 | 1964 | 155.0 | 57.3 | 108.9 | 71.9 | 26.7 | 1 | 35.5 | 53.8 | 85.8 | 41.1 | 32.0 | 53.1 |
| 1964_15 | 574813 | 6154663 | 1964 | 153.0 | 58.0 | 108.0 | 73.0 | 29.0 | 1 | 35.1 | 53.1 | 89.0 | 45.0 | 34.0 | 53.0 |
| 1964_10 | 574613 | 6154675 | 1964 | 152.0 | 61.0 | 112.5 | 73.5 | 29.0 | 1 | 39.5 | 53.9 | 91.0 | 46.5 | 36.5 | 52.6 |
| 1964_5 | 573800 | 6151450 | 1964 | 154.0 | 59.0 | 111.5 | 72.5 | 27.0 | 1 | 36.3 | 48.3 | 87.5 | 43.5 | 35.5 | 49.7 |
| 1964_11 | 573950 | 6151580 | 1964 | 153.7 | 58.1 | 110.1 | 71.9 | 27.2 | 1 | 36.3 | 49.3 | 90.0 | 42.7 | 34.6 | 49.6 |
| 1964_17 | 573311 | 6151519 | 1964 | 155.0 | 61.6 | 116.4 | 73.9 | 28.0 | 1 | 38.3 | 48.5 | 91.7 | 44.5 | 35.0 | 49.2 |
| 1964_16 | 573287 | 6151338 | 1964 | 156.0 | 66.0 | 119.0 | 73.0 | 29.0 | 1 | 36.0 | 52.6 | 97.0 | 45.0 | 35.0 | 50.2 |
| 1964_1 | 574000 | 6151450 | 1964 | 154.0 | 62.5 | 115.5 | 75.5 | 29.0 | 1 | 38.3 | 48.2 | 89.3 | 47.0 | 36.0 | 47.2 |
| 1964_4 | 573800 | 6151250 | 1964 | 154.8 | 64.0 | 118.8 | 74.0 | 29.5 | 1 | 40.0 | 48.2 | 95.3 | 45.8 | 35.5 | 49.7 |
| 1964_19 | 573590 | 6151478 | 1964 | 154.9 | 61.8 | 115.3 | 72.9 | 28.3 | 1 | 35.3 | 48.2 | 93.5 | 44.1 | 34.9 | 48.2 |
| 1964_2 | 574000 | 6151250 | 1964 | 155.0 | 66.0 | 120.3 | 75.0 | 29.8 | 1 | 41.3 | 50.6 | 97.3 | 48.3 | 37.0 | 49.4 |
| 1964_8 | 574811 | 6154286 | 1964 | 154.0 | 55.5 | 103.8 | 70.2 | 29.2 | 1 | 35.4 | 48.6 | 88.8 | 38.6 | 31.3 | 50.1 |
| 1964_9 | 574637 | 6154141 | 1964 | 153.0 | 59.0 | 109.0 | 71.0 | 29.0 | 1 | 36.3 | 49.0 | 90.0 | 43.0 | 35.0 | 48.7 |
| 1964_14 | 574818 | 6154861 | 1964 | 155.0 | 62.0 | 112.9 | 69.8 | 26.6 | 1 | 37.6 | 47.1 | 92.1 | 43.5 | 34.6 | 46.9 |
| 1964_6 | 574386 | 6154513 | 1964 | 154.0 | 70.0 | 122.0 | 77.9 | 30.0 | 1 | 41.0 | 54.0 | 97.9 | 54.6 | 39.9 | 55.2 |
| 1964_18 | 573455 | 6151611 | 1964 | 155.0 | 64.8 | 119.4 | 73.3 | 30.7 | 1 | 39.6 | 51.3 | 99.4 | 46.6 | 35.6 | 50.3 |
| 1964_7 | 574626 | 6154491 | 1964 | 154.9 | 63.7 | 116.3 | 71.9 | 28.9 | 1 | 34.8 | 50.1 | 95.5 | 45.8 | 35.3 | 50.0 |
| 1964_20 | 573736 | 6151280 | 1964 | 155.0 | 63.1 | 118.8 | 72.4 | 29.0 | 1 | 35.3 | 49.6 | 95.7 | 46.0 | 34.8 | 48.6 |
| 1964_13 | 574596 | 6154892 | 1964 | 156.0 | 75.0 | 130.7 | 78.3 | 33.5 | 1 | 43.2 | 55.8 | 111.7 | 53.0 | 40.0 | 56.8 |
| 1964_12 | 574400 | 6154998 | 1964 | 156.0 | 74.5 | 129.6 | 78.2 | 34.9 | 1 | 42.1 | 51.3 | 109.1 | 55.2 | 40.2 | 52.9 |
| B11S10 | 505816 | 6159491 | 1965 | 145.0 | 56.0 | 104.3 | 64.4 | 31.8 | 2 | 39.3 | 59.4 | 97.4 | 38.9 | 30.3 | 54.7 |
| B02S20 | 609015 | 6152502 | 1966 | 156.0 | 70.3 | 125.1 | 72.2 | 30.7 | 1 | 39.5 | 64.6 | 101.7 | 49.3 | 37.1 | 62.9 |
| M17S02 | 451989 | 6127523 | 1966 | 143.9 | 53.4 | 99.7 | 72.8 | 29.4 | 3 | 40.6 | 75.8 | 81.8 | 41.9 | 35.9 | 68.7 |
| B13S15 | 438023 | 6265991 | 1966 | 151.0 | 70.6 | 124.1 | 70.9 | 31.4 | 2 | 41.4 | 53.2 | 110.2 | 52.4 | 35.8 | 53.5 |
| B03S05 | 589752 | 6153504 | 1966 | 153.0 | 71.9 | 127.7 | 79.0 | 30.3 | 1 | 40.5 | 53.2 | 97.0 | 54.7 | 40.2 | 54.9 |
| M06S07 | 569170 | 6158833 | 1966 | 156.8 | 69.3 | 125.4 | 78.5 | 30.1 | 1 | 40.2 | 50.0 | 102.9 | 50.1 | 39.2 | 49.6 |
| B15S08 | 426010 | 6265406 | 1966 | 150.0 | 67.9 | 123.9 | 77.0 | 33.3 | 2 | 38.0 | 63.9 | 108.5 | 53.6 | 38.3 | 62.7 |
| B17S12 | 451501 | 6127470 | 1967 | 144.9 | 47.8 | 93.1 | 69.0 | 27.1 | 3 | 38.9 | 53.3 | 81.7 | 35.8 | 31.8 | 45.4 |
| M15S04 | 426848 | 6265469 | 1967 | 151.0 | 65.3 | 119.3 | 73.6 | 31.3 | 2 | 37.6 | 54.8 | 99.5 | 46.9 | 35.1 | 58.3 |
| M05S05 | 569167 | 6173427 | 1967 | 157.0 | 66.7 | 124.3 | 72.6 | 29.8 | 2 | 37.5 | 53.2 | 106.4 | 49.2 | 37.0 | 50.5 |
| B14S12 | 426477 | 6258995 | 1967 | 148.0 | 67.8 | 123.3 | 74.5 | 29.7 | 1 | 37.9 | 54.6 | 100.0 | 52.4 | 37.8 | 51.5 |
| M13S10 | 438329 | 6265416 | 1968 | 150.0 | 67.2 | 122.1 | 75.6 | 29.8 | 1 | 42.6 | 47.3 | 103.4 | 52.0 | 36.5 | 52.0 |
| M13S07 | 437445 | 6265395 | 1968 | 155.0 | 69.3 | 123.2 | 73.4 | 32.6 | 1 | 44.1 | 49.8 | 110.7 | 52.8 | 36.7 | 52.1 |
| B15S11 | 427008 | 6266846 | 1968 | 152.8 | 67.2 | 122.6 | 69.8 | 30.1 | 2 | 35.7 | 62.7 | 102.1 | 48.9 | 35.4 | 61.4 |
| OLD_B15S02 | 427504 | 6264892 | 1968 | 160.2 | 85.7 | 139.1 | 79.3 | 28.8 | 1 | 34.8 | 51.5 | 100.1 | 61.4 | 40.4 | 56.1 |
| B02S03 | 607485 | 6151009 | 1969 | 155.9 | 62.2 | 112.4 | 71.1 | 27.1 | 1 | 37.0 | 54.8 | 89.7 | 45.8 | 35.0 | 55.0 |
| B16S16 | 412005 | 6269992 | 1969 | 149.1 | 61.4 | 111.0 | 72.4 | 27.4 | 1 | 33.2 | 56.1 | 95.0 | 46.1 | 34.7 | 56.1 |
| M13S09 | 437968 | 6265311 | 1969 | 152.0 | 75.5 | 132.1 | 77.5 | 32.9 | 1 | 44.7 | 52.1 | 110.8 | 55.5 | 38.2 | 56.0 |
| B14S03 | 426482 | 6260010 | 1969 | 150.0 | 76.4 | 132.6 | 76.8 | 31.7 | 2 | 36.8 | 62.1 | 106.1 | 56.7 | 39.5 | 60.8 |
| B14S11 | 427024 | 6259014 | 1970 | 151.5 | 67.3 | 123.3 | 73.1 | 30.5 | 2 | 36.5 | 53.1 | 98.9 | 52.6 | 36.1 | 56.7 |
| M14S12 | 427036 | 6258596 | 1970 | 149.0 | 68.5 | 126.7 | 73.0 | 30.7 | 2 | 37.0 | 52.6 | 105.1 | 51.1 | 37.9 | 54.1 |
| M15S03 | 427971 | 6265120 | 1971 | 151.0 | 70.3 | 128.9 | 75.0 | 31.2 | 2 | 36.6 | 64.5 | 100.1 | 52.7 | 36.9 | 63.3 |
| M07S06 | 562442 | 6158010 | 1971 | 156.0 | 73.3 | 131.6 | 77.4 | 32.3 | 1 | 42.2 | 57.8 | 108.4 | 57.1 | 39.9 | 58.9 |
| B10S36 | 541474 | 6156789 | 1972 | 149.0 | 56.5 | 108.7 | 66.8 | 24.6 | 1 | 36.1 | 60.5 | 73.6 | 41.4 | 33.3 | 59.5 |
| B09S20 | 545993 | 6167008 | 1972 | 155.0 | 69.3 | 121.3 | 76.2 | 33.2 | 1 | 43.1 | 65.0 | 95.4 | 51.6 | 38.3 | 63.8 |
| M10S05 | 544555 | 6157163 | 1972 | 156.0 | 65.4 | 115.0 | 76.0 | 33.3 | 1 | 44.4 | 64.0 | 99.4 | 48.0 | 36.0 | 61.0 |
| B25S10 | 652500 | 6205001 | 1972 | 169.7 | 69.4 | 132.9 | 79.5 | 28.5 | 1 | 34.7 | 58.5 | 98.2 | 50.2 | 37.7 | 56.4 |
| B13S20 | 437576 | 6265064 | 1972 | 150.0 | 63.2 | 119.8 | 72.5 | 31.7 | 2 | 45.0 | 50.5 | 110.7 | 46.5 | 34.9 | 50.0 |
| B08S08 | 546003 | 6175507 | 1972 | 150.0 | 53.2 | 103.3 | 65.0 | 28.9 | 2 | 38.0 | 53.3 | 84.1 | 39.0 | 29.9 | 52.2 |
| B13S13 | 437522 | 6266472 | 1972 | 149.0 | 68.9 | 124.3 | 73.6 | 35.0 | 2 | 45.8 | 61.0 | 112.0 | 55.1 | 38.1 | 59.5 |
| M05S02 | 568589 | 6175108 | 1972 | 156.0 | 61.7 | 113.9 | 71.1 | 30.1 | 2 | 40.4 | 52.1 | 102.2 | 46.4 | 35.1 | 50.2 |
| B13S18 | 437995 | 6264476 | 1972 | 151.4 | 67.5 | 122.0 | 75.4 | 33.5 | 2 | 44.0 | 55.7 | 113.4 | 51.8 | 37.5 | 56.7 |
| OLD_B13S20 | 437500 | 6265005 | 1972 | 151.0 | 67.1 | 121.1 | 73.3 | 32.0 | 2 | 40.5 | 51.3 | 104.1 | 49.5 | 36.1 | 54.0 |
| M14S11 | 426994 | 6257705 | 1972 | 149.3 | 62.2 | 114.0 | 74.4 | 28.0 | 1 | 36.0 | 51.4 | 93.7 | 46.7 | 36.0 | 51.2 |
| M13S08 | 437674 | 6265279 | 1972 | 152.0 | 74.0 | 129.1 | 76.3 | 29.8 | 1 | 42.0 | 58.5 | 99.3 | 53.0 | 36.6 | 59.4 |
| B15S06 | 426980 | 6265408 | 1972 | 150.1 | 69.7 | 125.9 | 75.9 | 30.6 | 2 | 38.4 | 55.7 | 98.6 | 51.2 | 37.8 | 58.5 |
| B11S05 | 507525 | 6159498 | 1972 | 150.2 | 56.1 | 105.5 | 69.3 | 28.1 | 1 | 38.3 | 55.4 | 86.0 | 40.6 | 33.2 | 48.2 |
| B05S02 | 568498 | 6175000 | 1972 | 156.0 | 65.7 | 117.7 | 72.1 | 31.0 | 2 | 39.7 | 50.0 | 103.1 | 47.5 | 35.7 | 49.8 |
| B15S12 | 427568 | 6267035 | 1972 | 148.0 | 62.8 | 111.2 | 71.3 | 30.4 | 2 | 37.4 | 58.1 | 100.6 | 47.7 | 34.9 | 57.9 |
| B11S04 | 507519 | 6158970 | 1972 | 150.3 | 62.2 | 113.2 | 70.9 | 33.1 | 1 | 43.1 | 58.7 | 103.4 | 44.6 | 35.0 | 54.1 |
| B05S18 | 568021 | 6174005 | 1972 | 156.0 | 65.0 | 121.2 | 71.5 | 30.3 | 2 | 38.9 | 51.2 | 101.9 | 48.9 | 35.5 | 50.8 |
| M11S08 | 505699 | 6157670 | 1972 | 148.3 | 55.5 | 104.8 | 67.6 | 31.7 | 1 | 43.2 | 56.5 | 99.0 | 38.9 | 30.3 | 54.5 |
| M16S09 | 411577 | 6270565 | 1972 | 148.0 | 63.5 | 116.9 | 71.6 | 28.6 | 2 | 35.4 | 51.8 | 96.7 | 48.5 | 36.0 | 56.0 |
| B15S07 | 426508 | 6265394 | 1972 | 150.0 | 62.4 | 115.7 | 72.3 | 28.4 | 2 | 35.1 | 50.8 | 96.9 | 47.6 | 35.3 | 57.9 |
| B05S03 | 568033 | 6175006 | 1972 | 156.1 | 64.7 | 118.7 | 73.9 | 31.1 | 2 | 38.0 | 51.1 | 107.4 | 47.7 | 35.9 | 49.7 |
| M07S05 | 562459 | 6158264 | 1972 | 157.0 | 67.8 | 125.1 | 73.6 | 30.4 | 1 | 37.2 | 58.4 | 101.0 | 53.1 | 37.4 | 57.5 |
| B05S01 | 569003 | 6174999 | 1972 | 156.3 | 68.3 | 121.8 | 74.4 | 30.1 | 2 | 38.2 | 55.7 | 103.3 | 50.5 | 35.8 | 54.7 |
| B16S07 | 411986 | 6268519 | 1972 | 149.0 | 61.3 | 118.0 | 72.0 | 31.1 | 2 | 36.3 | 56.5 | 102.2 | 48.5 | 35.4 | 54.5 |
| B13S12 | 437525 | 6266006 | 1972 | 149.0 | 71.7 | 126.6 | 73.1 | 33.0 | 2 | 42.1 | 56.2 | 113.4 | 54.9 | 37.6 | 58.4 |
| B05S04 | 567977 | 6175512 | 1972 | 157.0 | 63.0 | 118.1 | 72.1 | 29.6 | 2 | 36.6 | 51.1 | 103.8 | 46.1 | 35.1 | 49.1 |
| B13S19 | 437587 | 6264609 | 1972 | 153.2 | 70.9 | 125.4 | 75.1 | 32.2 | 1 | 46.0 | 46.0 | 106.8 | 52.8 | 38.0 | 50.5 |
| B15S19 | 426535 | 6267506 | 1972 | 150.0 | 61.6 | 111.9 | 72.3 | 29.2 | 1 | 37.7 | 49.6 | 96.0 | 47.7 | 35.7 | 51.4 |
| M15S07 | 426880 | 6267662 | 1972 | 149.7 | 61.6 | 113.5 | 72.3 | 29.0 | 2 | 34.3 | 54.8 | 97.9 | 47.9 | 35.3 | 56.9 |
| M15S06 | 426942 | 6266082 | 1972 | 149.8 | 66.4 | 118.1 | 71.7 | 29.6 | 2 | 36.7 | 58.6 | 100.0 | 49.2 | 35.3 | 60.5 |
| B15S05 | 427510 | 6265419 | 1972 | 151.3 | 66.9 | 122.1 | 75.9 | 30.8 | 2 | 36.4 | 56.0 | 101.8 | 51.9 | 36.7 | 60.9 |
| B26S11 | 646963 | 6210469 | 1972 | 161.0 | 61.0 | 113.0 | 68.3 | 26.8 | 2 | 36.3 | 58.5 | 98.0 | 42.3 | 32.0 | 58.5 |
| B05S05 | 567974 | 6175988 | 1972 | 156.0 | 68.0 | 124.3 | 73.1 | 29.4 | 2 | 38.1 | 59.9 | 105.3 | 49.1 | 36.1 | 58.0 |
| B15S14 | 427989 | 6267519 | 1972 | 148.0 | 63.6 | 117.6 | 73.2 | 29.0 | 1 | 32.3 | 52.8 | 98.1 | 48.0 | 35.0 | 54.2 |
| M03S08 | 589351 | 6153242 | 1972 | 154.0 | 70.6 | 129.1 | 76.4 | 28.9 | 1 | 40.3 | 51.1 | 98.1 | 51.8 | 39.4 | 50.0 |
| B15S04 | 428104 | 6265364 | 1972 | 150.0 | 68.6 | 125.0 | 73.9 | 31.7 | 2 | 37.6 | 58.3 | 106.0 | 50.6 | 36.7 | 58.5 |
| B13S14 | 437987 | 6266520 | 1972 | 152.0 | 75.7 | 133.8 | 75.7 | 35.7 | 1 | 49.4 | 51.6 | 119.5 | 56.4 | 39.0 | 54.0 |
| B15S18 | 426525 | 6267994 | 1972 | 149.0 | 61.5 | 115.4 | 71.0 | 27.5 | 1 | 35.0 | 48.7 | 97.9 | 45.9 | 34.0 | 50.9 |
| B05S06 | 568472 | 6176000 | 1972 | 156.2 | 70.7 | 127.2 | 74.3 | 30.9 | 2 | 38.4 | 51.4 | 106.6 | 51.7 | 37.6 | 51.1 |
| B05S20 | 568472 | 6173517 | 1972 | 155.0 | 59.1 | 114.9 | 69.5 | 26.4 | 2 | 34.2 | 51.4 | 99.3 | 43.9 | 32.7 | 51.9 |
| M04S05 | 588927 | 6147127 | 1972 | 152.6 | 72.1 | 127.2 | 77.8 | 30.9 | 1 | 41.5 | 44.5 | 105.6 | 52.9 | 39.4 | 44.5 |
| B05S07 | 569000 | 6176002 | 1972 | 155.6 | 64.7 | 121.5 | 71.5 | 31.9 | 2 | 40.1 | 51.1 | 113.1 | 49.4 | 35.6 | 54.0 |
| B15S16 | 427417 | 6267854 | 1972 | 149.0 | 64.1 | 115.4 | 74.0 | 29.8 | 1 | 36.8 | 50.5 | 98.7 | 50.0 | 36.0 | 51.3 |
| B26S18 | 645993 | 6211122 | 1972 | 159.5 | 65.2 | 118.0 | 69.0 | 27.9 | 2 | 36.2 | 56.2 | 99.5 | 44.7 | 32.7 | 55.8 |
| M16S07 | 411838 | 6269786 | 1972 | 148.0 | 69.7 | 121.7 | 74.9 | 30.9 | 1 | 38.9 | 56.7 | 102.0 | 53.8 | 36.0 | 56.3 |
| B08S09 | 546518 | 6175502 | 1972 | 151.0 | 59.1 | 110.2 | 67.8 | 31.7 | 1 | 41.6 | 51.3 | 94.6 | 43.7 | 32.8 | 51.6 |
| M08S06 | 545548 | 6172932 | 1972 | 153.8 | 65.5 | 119.6 | 64.6 | 32.3 | 2 | 40.8 | 56.0 | 108.7 | 44.9 | 32.5 | 56.5 |
| B13S11 | 437599 | 6265489 | 1972 | 152.5 | 70.7 | 125.3 | 71.4 | 31.0 | 1 | 44.0 | 47.1 | 105.5 | 51.4 | 36.9 | 51.1 |
| M15S10 | 426960 | 6268462 | 1972 | 150.0 | 69.5 | 122.2 | 71.1 | 29.9 | 2 | 35.9 | 67.3 | 97.7 | 54.3 | 36.9 | 67.6 |
| B20S19 | 489935 | 6338478 | 1972 | 155.0 | 79.3 | 134.9 | 73.5 | 33.5 | 2 | 44.0 | 57.0 | 117.7 | 56.0 | 37.9 | 58.8 |
| B24S09 | 510437 | 6309519 | 1972 | 153.0 | 75.2 | 128.1 | 69.3 | 31.8 | 1 | 42.8 | 59.4 | 110.0 | 54.7 | 36.0 | 61.4 |
| M20S07 | 490653 | 6338140 | 1972 | 155.0 | 78.4 | 132.4 | 73.8 | 33.6 | 2 | 42.3 | 52.0 | 112.5 | 59.2 | 38.5 | 55.9 |
| M20S09 | 490619 | 6338875 | 1972 | 154.5 | 77.5 | 131.0 | 71.5 | 32.3 | 2 | 41.9 | 52.7 | 113.3 | 56.3 | 37.5 | 55.0 |
| M05S01 | 568636 | 6175375 | 1972 | 157.0 | 79.1 | 139.8 | 78.9 | 35.4 | 2 | 43.9 | 58.8 | 120.1 | 59.2 | 41.4 | 57.6 |
| M26S09 | 646517 | 6208393 | 1972 | 162.0 | 66.9 | 118.1 | 68.4 | 28.2 | 2 | 34.9 | 61.3 | 98.4 | 47.7 | 34.1 | 59.8 |
| B06S08 | 567539 | 6158005 | 1972 | 156.0 | 73.7 | 128.4 | 75.4 | 30.7 | 1 | 37.8 | 48.2 | 102.2 | 51.7 | 37.5 | 48.6 |
| B05S19 | 568006 | 6173510 | 1972 | 157.0 | 79.5 | 140.0 | 77.7 | 36.0 | 2 | 42.9 | 58.4 | 121.3 | 60.7 | 41.0 | 57.5 |
| M20S05 | 490661 | 6337442 | 1972 | 156.0 | 81.3 | 137.7 | 70.9 | 31.8 | 2 | 41.7 | 58.4 | 113.6 | 58.1 | 36.8 | 59.0 |
| M20S04 | 490562 | 6337130 | 1972 | 157.0 | 83.7 | 139.4 | 72.1 | 33.3 | 2 | 41.6 | 51.2 | 115.9 | 60.2 | 37.7 | 55.6 |
| B20S10 | 490603 | 6336998 | 1972 | 158.0 | 88.1 | 146.0 | 70.5 | 35.7 | 2 | 43.4 | 65.6 | 131.2 | 62.8 | 38.3 | 66.9 |
| M27S02 | 648307 | 6257763 | 1975 | 156.2 | 57.3 | 105.1 | 71.4 | 28.2 | 1 | 37.2 | 80.8 | 93.6 | 43.7 | 33.7 | 77.6 |
| M27S03 | 648587 | 6257523 | 1975 | 158.0 | 65.1 | 118.1 | 72.0 | 31.2 | 1 | 40.3 | 82.2 | 103.1 | 46.1 | 34.0 | 77.5 |
| B27S18 | 648503 | 6258502 | 1975 | 157.6 | 64.2 | 115.3 | 72.5 | 30.2 | 1 | 38.4 | 76.1 | 101.4 | 45.9 | 35.2 | 73.7 |
| B27S20 | 648457 | 6257500 | 1975 | 158.0 | 64.4 | 116.0 | 71.1 | 29.4 | 1 | 39.3 | 81.7 | 102.7 | 46.3 | 35.1 | 77.2 |
| B28S34 | 661003 | 6281003 | 1975 | 159.6 | 65.6 | 119.0 | 73.7 | 27.8 | 1 | 36.9 | 73.3 | 96.3 | 48.6 | 36.1 | 71.5 |
| B27S16 | 648885 | 6259004 | 1975 | 158.0 | 66.6 | 120.0 | 72.9 | 29.4 | 1 | 40.3 | 75.5 | 105.5 | 49.5 | 36.1 | 71.5 |
| B27S19 | 648504 | 6258011 | 1975 | 159.9 | 65.4 | 118.5 | 71.0 | 29.6 | 1 | 38.9 | 75.0 | 103.3 | 46.4 | 35.4 | 75.8 |
| M28S05 | 661071 | 6280459 | 1975 | 159.1 | 55.6 | 104.5 | 70.6 | 26.8 | 1 | 34.9 | 69.4 | 91.4 | 41.7 | 31.6 | 68.7 |
| B28S05 | 662522 | 6283003 | 1975 | 158.9 | 67.4 | 118.1 | 72.4 | 28.9 | 1 | 36.6 | 75.2 | 96.0 | 50.0 | 35.5 | 71.1 |
| M27S04 | 648720 | 6257321 | 1975 | 158.0 | 71.8 | 127.2 | 73.0 | 30.1 | 1 | 39.8 | 78.9 | 104.0 | 53.5 | 37.8 | 75.7 |
| M28S08 | 661536 | 6279973 | 1975 | 159.5 | 65.5 | 119.2 | 72.3 | 29.6 | 1 | 37.6 | 82.0 | 106.3 | 47.9 | 36.3 | 78.1 |
| M25S06 | 651716 | 6205879 | 1975 | 169.0 | 68.4 | 125.5 | 77.2 | 27.0 | 1 | 36.3 | 67.4 | 91.9 | 47.7 | 36.3 | 68.1 |
| B27S13 | 649008 | 6258475 | 1975 | 159.4 | 65.8 | 121.7 | 73.7 | 30.4 | 1 | 37.6 | 71.6 | 101.8 | 47.7 | 35.5 | 71.0 |
| M25S03 | 650285 | 6205614 | 1975 | 167.0 | 64.3 | 118.2 | 79.0 | 26.0 | 1 | 34.0 | 65.8 | 85.2 | 48.3 | 36.9 | 66.7 |
| M27S01 | 647873 | 6258574 | 1975 | 159.0 | 65.1 | 117.7 | 71.6 | 28.5 | 1 | 35.5 | 71.4 | 97.2 | 47.8 | 35.0 | 69.2 |
| B25S19 | 651478 | 6204751 | 1975 | 168.0 | 71.5 | 129.8 | 78.4 | 26.7 | 1 | 35.7 | 67.5 | 91.5 | 48.8 | 37.2 | 68.4 |
| B28S08 | 661523 | 6282501 | 1975 | 158.0 | 66.3 | 121.1 | 72.7 | 30.7 | 1 | 38.0 | 73.6 | 105.0 | 49.5 | 36.2 | 72.6 |
| B27S12 | 649020 | 6258004 | 1975 | 159.9 | 68.1 | 123.0 | 72.5 | 29.3 | 1 | 38.0 | 73.1 | 105.0 | 48.1 | 36.5 | 69.0 |
| B27S17 | 648498 | 6258999 | 1975 | 158.0 | 68.0 | 121.6 | 71.4 | 30.1 | 1 | 37.5 | 78.5 | 107.7 | 49.2 | 36.0 | 77.8 |
| B28S10 | 660489 | 6282509 | 1975 | 161.0 | 73.4 | 128.3 | 76.7 | 29.7 | 1 | 36.9 | 73.8 | 101.3 | 54.4 | 37.9 | 72.2 |
| B28S33 | 661001 | 6280503 | 1975 | 160.5 | 66.9 | 122.5 | 74.2 | 29.3 | 1 | 36.0 | 71.3 | 100.4 | 48.2 | 35.3 | 68.5 |
| B25S04 | 651992 | 6206498 | 1975 | 168.0 | 71.1 | 130.5 | 78.6 | 27.8 | 1 | 35.2 | 66.7 | 92.2 | 48.8 | 37.1 | 68.5 |
| B28S06 | 662495 | 6282499 | 1975 | 159.0 | 71.8 | 123.7 | 72.9 | 30.8 | 1 | 36.7 | 72.8 | 99.6 | 51.4 | 36.7 | 70.4 |
| B28S03 | 661473 | 6282988 | 1975 | 160.0 | 70.5 | 124.4 | 71.5 | 30.4 | 1 | 37.8 | 72.5 | 103.4 | 49.3 | 35.5 | 69.1 |
| B28S09 | 661020 | 6282489 | 1975 | 158.7 | 70.8 | 126.1 | 72.8 | 29.1 | 1 | 36.5 | 73.0 | 102.2 | 52.0 | 36.6 | 68.6 |
| B28S20 | 661502 | 6280504 | 1975 | 159.4 | 74.5 | 129.0 | 74.4 | 30.1 | 1 | 38.2 | 73.0 | 107.4 | 52.3 | 37.1 | 72.0 |
| B25S07 | 652016 | 6205000 | 1975 | 169.0 | 72.4 | 131.1 | 81.9 | 27.6 | 1 | 34.5 | 69.2 | 93.2 | 51.6 | 39.0 | 67.3 |
| B28S07 | 662003 | 6282476 | 1975 | 160.0 | 66.0 | 118.8 | 72.2 | 31.0 | 1 | 38.7 | 71.6 | 106.4 | 49.8 | 36.2 | 69.3 |
| B27S11 | 649019 | 6257511 | 1975 | 159.0 | 67.2 | 121.5 | 72.0 | 29.7 | 1 | 35.3 | 70.6 | 106.5 | 47.4 | 35.1 | 70.2 |
| M28S02 | 660171 | 6282126 | 1975 | 161.0 | 65.1 | 118.9 | 71.8 | 35.3 | 1 | 40.3 | 79.0 | 120.7 | 48.5 | 35.2 | 77.1 |
| B25S06 | 651979 | 6205399 | 1975 | 166.0 | 64.6 | 123.3 | 80.3 | 26.8 | 1 | 34.7 | 65.8 | 87.2 | 46.7 | 37.3 | 65.4 |
| M25S05 | 651555 | 6205974 | 1975 | 167.0 | 69.3 | 126.4 | 80.0 | 26.5 | 1 | 34.9 | 62.8 | 89.3 | 47.3 | 37.2 | 67.2 |
| B28S19 | 661551 | 6280846 | 1975 | 159.3 | 73.4 | 127.8 | 73.3 | 31.7 | 1 | 38.8 | 73.8 | 110.3 | 52.2 | 37.6 | 71.7 |
| B26S08 | 645754 | 6209500 | 1975 | 159.0 | 65.7 | 116.9 | 70.8 | 29.2 | 1 | 36.8 | 67.2 | 102.5 | 47.8 | 35.2 | 66.5 |
| B26S10 | 646221 | 6209010 | 1975 | 161.0 | 64.7 | 116.1 | 69.4 | 27.7 | 1 | 36.9 | 64.5 | 98.7 | 44.4 | 32.8 | 63.9 |
| M28S03 | 660133 | 6281905 | 1975 | 160.0 | 74.0 | 127.3 | 73.9 | 35.8 | 1 | 44.2 | 78.3 | 122.3 | 52.1 | 37.0 | 77.3 |
| B25S18 | 651011 | 6204729 | 1975 | 167.4 | 65.7 | 124.6 | 78.6 | 26.4 | 1 | 35.6 | 62.6 | 87.8 | 45.6 | 36.0 | 64.3 |
| B28S04 | 661985 | 6283020 | 1975 | 160.6 | 71.2 | 126.5 | 72.3 | 30.4 | 1 | 37.8 | 68.6 | 105.2 | 50.7 | 36.6 | 68.1 |
| B25S05 | 651998 | 6205999 | 1975 | 169.2 | 71.2 | 130.3 | 79.5 | 27.8 | 1 | 35.1 | 66.6 | 91.7 | 51.2 | 38.3 | 65.1 |
| B26S09 | 646254 | 6209502 | 1975 | 156.4 | 55.7 | 102.2 | 67.2 | 25.0 | 1 | 31.0 | 62.9 | 79.4 | 40.9 | 30.9 | 65.0 |
| B25S17 | 650978 | 6205227 | 1975 | 167.0 | 67.8 | 125.0 | 78.5 | 26.6 | 1 | 33.5 | 63.8 | 89.7 | 49.6 | 36.8 | 64.5 |
| M25S02 | 649753 | 6205390 | 1975 | 165.0 | 73.2 | 129.9 | 80.4 | 27.4 | 1 | 36.2 | 62.9 | 93.3 | 51.3 | 37.5 | 67.8 |
| B25S13 | 651020 | 6206226 | 1975 | 169.5 | 63.2 | 118.6 | 78.2 | 26.2 | 1 | 34.6 | 61.4 | 85.2 | 45.8 | 35.5 | 63.6 |
| B11S11 | 506003 | 6158028 | 1975 | 149.0 | 55.2 | 102.1 | 70.1 | 28.4 | 1 | 40.8 | 69.2 | 85.0 | 42.2 | 33.8 | 63.0 |
| B11S19 | 505484 | 6158530 | 1975 | 149.0 | 58.8 | 103.5 | 69.3 | 31.1 | 1 | 41.9 | 69.8 | 88.1 | 42.5 | 34.3 | 64.3 |
| M28S06 | 661289 | 6280217 | 1975 | 160.0 | 72.2 | 126.2 | 75.5 | 34.9 | 1 | 40.8 | 76.6 | 116.0 | 54.7 | 38.0 | 72.3 |
| B26S06 | 646250 | 6209998 | 1975 | 157.0 | 50.2 | 95.7 | 67.3 | 24.4 | 1 | 34.0 | 62.8 | 78.2 | 35.4 | 30.3 | 62.0 |
| B25S03 | 652499 | 6206501 | 1975 | 169.5 | 75.2 | 136.4 | 79.5 | 28.5 | 1 | 36.8 | 65.9 | 99.2 | 51.3 | 38.3 | 65.6 |
| B26S17 | 646018 | 6211486 | 1975 | 158.8 | 55.3 | 103.5 | 67.8 | 26.0 | 1 | 32.2 | 61.4 | 86.5 | 39.6 | 31.6 | 62.0 |
| B26S20 | 646516 | 6210496 | 1975 | 159.0 | 56.6 | 103.4 | 67.7 | 26.2 | 1 | 34.6 | 61.3 | 85.1 | 38.7 | 31.1 | 61.2 |
| B26S07 | 645740 | 6209996 | 1975 | 157.4 | 54.7 | 101.1 | 66.9 | 24.4 | 1 | 32.7 | 58.9 | 83.5 | 37.8 | 30.9 | 61.5 |
| B25S12 | 651482 | 6206267 | 1975 | 168.0 | 66.2 | 121.8 | 79.9 | 26.0 | 1 | 31.7 | 60.8 | 84.0 | 46.2 | 36.6 | 60.6 |
| M05S10 | 569471 | 6172883 | 1975 | 156.0 | 68.8 | 123.6 | 75.2 | 31.7 | 1 | 38.8 | 67.1 | 108.0 | 51.0 | 36.2 | 68.0 |
| M26S07 | 646345 | 6209605 | 1975 | 157.9 | 61.4 | 107.8 | 66.6 | 26.4 | 1 | 33.3 | 60.6 | 90.5 | 43.3 | 31.9 | 60.9 |
| B11S17 | 506501 | 6158505 | 1975 | 150.3 | 63.2 | 110.9 | 71.1 | 32.0 | 1 | 41.5 | 67.6 | 91.2 | 43.8 | 34.3 | 63.6 |
| B03S01 | 589903 | 6154485 | 1975 | 153.0 | 72.9 | 127.4 | 75.9 | 30.5 | 1 | 40.4 | 67.7 | 107.7 | 51.8 | 38.1 | 68.6 |
| B25S20 | 651477 | 6205235 | 1975 | 167.9 | 65.6 | 122.9 | 77.5 | 26.6 | 1 | 34.6 | 60.2 | 86.8 | 47.9 | 36.5 | 61.4 |
| B28S37 | 662003 | 6281498 | 1975 | 158.2 | 68.1 | 121.7 | 71.0 | 31.1 | 1 | 39.5 | 67.4 | 105.4 | 50.2 | 36.3 | 64.5 |
| B03S02 | 589403 | 6154522 | 1975 | 154.2 | 67.6 | 120.2 | 77.3 | 30.2 | 1 | 39.6 | 66.0 | 96.7 | 50.8 | 38.9 | 66.4 |
| B26S01 | 646739 | 6208998 | 1975 | 160.6 | 67.2 | 116.2 | 69.6 | 28.0 | 1 | 36.0 | 59.8 | 98.3 | 45.3 | 34.6 | 62.4 |
| B05S11 | 569500 | 6173002 | 1975 | 155.3 | 68.2 | 123.7 | 74.5 | 31.2 | 1 | 37.7 | 66.3 | 102.8 | 49.8 | 36.6 | 65.5 |
| B11S18 | 506003 | 6158500 | 1975 | 150.4 | 62.8 | 109.3 | 73.9 | 31.6 | 1 | 44.6 | 66.5 | 91.1 | 44.3 | 34.8 | 64.4 |
| M26S08 | 646628 | 6208963 | 1975 | 161.0 | 64.4 | 112.6 | 69.0 | 27.6 | 1 | 36.6 | 60.1 | 97.4 | 43.6 | 33.6 | 60.2 |
| B11S09 | 505783 | 6160014 | 1975 | 147.1 | 54.8 | 105.9 | 70.3 | 28.0 | 1 | 38.8 | 64.9 | 95.2 | 42.1 | 32.2 | 59.2 |
| B28S02 | 661007 | 6282978 | 1975 | 160.5 | 80.4 | 138.1 | 75.1 | 34.4 | 1 | 41.6 | 76.5 | 124.6 | 59.1 | 39.0 | 70.3 |
| B25S02 | 652497 | 6205996 | 1975 | 170.2 | 75.5 | 135.0 | 80.5 | 27.9 | 1 | 37.8 | 64.0 | 97.8 | 53.1 | 39.8 | 62.7 |
| M25S07 | 652045 | 6205742 | 1975 | 168.2 | 74.8 | 133.5 | 80.3 | 27.4 | 1 | 36.4 | 61.7 | 97.5 | 53.1 | 38.4 | 62.3 |
| B11S12 | 506478 | 6158008 | 1975 | 151.0 | 65.2 | 114.1 | 75.5 | 33.5 | 1 | 47.0 | 70.0 | 95.5 | 48.6 | 37.2 | 65.6 |
| M26S05 | 646405 | 6209984 | 1975 | 156.0 | 47.4 | 90.4 | 66.7 | 22.0 | 1 | 30.7 | 58.7 | 74.5 | 34.4 | 29.7 | 57.2 |
| B11S08 | 505249 | 6160032 | 1975 | 148.0 | 62.8 | 111.6 | 68.1 | 28.3 | 1 | 38.3 | 61.6 | 87.0 | 43.7 | 33.8 | 59.3 |
| B25S14 | 650515 | 6206249 | 1975 | 168.0 | 68.4 | 127.4 | 78.1 | 26.5 | 1 | 32.8 | 59.9 | 88.1 | 48.6 | 36.9 | 57.0 |
| M11S06 | 505678 | 6158534 | 1975 | 150.0 | 58.5 | 108.2 | 71.1 | 32.2 | 1 | 44.2 | 67.3 | 89.6 | 41.8 | 35.1 | 60.3 |
| B03S03 | 589619 | 6154986 | 1975 | 152.8 | 57.8 | 110.6 | 74.6 | 29.9 | 1 | 39.2 | 61.2 | 97.2 | 45.6 | 35.4 | 61.2 |
| M11S07 | 505686 | 6158248 | 1975 | 149.0 | 65.8 | 114.1 | 74.7 | 33.0 | 1 | 43.0 | 68.3 | 92.4 | 48.1 | 38.0 | 63.3 |
| B28S01 | 660529 | 6282988 | 1975 | 158.3 | 77.9 | 135.2 | 73.3 | 31.4 | 1 | 38.7 | 68.1 | 109.4 | 59.6 | 39.0 | 66.0 |
| B11S20 | 505490 | 6158023 | 1975 | 148.0 | 56.8 | 102.5 | 69.2 | 29.0 | 1 | 38.7 | 61.2 | 82.5 | 40.5 | 33.0 | 57.1 |
| M03S06 | 589400 | 6154181 | 1975 | 153.6 | 71.1 | 123.1 | 77.5 | 31.1 | 1 | 41.6 | 63.5 | 100.8 | 52.5 | 39.1 | 66.0 |
| B26S19 | 646020 | 6210497 | 1975 | 160.0 | 62.9 | 113.1 | 69.2 | 28.4 | 1 | 36.5 | 58.7 | 94.2 | 45.5 | 33.5 | 56.2 |
| B25S01 | 652599 | 6205597 | 1975 | 169.0 | 79.4 | 142.4 | 81.8 | 29.7 | 1 | 39.0 | 62.2 | 103.2 | 56.5 | 40.6 | 66.6 |
| B05S12 | 570006 | 6172997 | 1975 | 155.0 | 61.9 | 117.8 | 71.2 | 29.9 | 1 | 36.1 | 62.0 | 99.6 | 45.0 | 34.7 | 57.5 |
| M26S04 | 646606 | 6209919 | 1975 | 158.8 | 63.4 | 114.4 | 72.5 | 31.2 | 1 | 40.3 | 58.9 | 98.1 | 46.7 | 34.8 | 58.4 |
| B12S04 | 502021 | 6165444 | 1975 | 150.0 | 67.9 | 122.4 | 74.2 | 35.0 | 2 | 45.9 | 63.8 | 100.6 | 52.1 | 38.0 | 60.5 |
| M12S03 | 499590 | 6167222 | 1975 | 149.0 | 75.3 | 130.6 | 78.7 | 35.9 | 2 | 46.5 | 60.4 | 106.3 | 57.9 | 41.5 | 57.4 |
| B12S07 | 500476 | 6165495 | 1975 | 149.0 | 59.6 | 110.4 | 68.5 | 29.9 | 2 | 41.9 | 56.5 | 94.9 | 40.9 | 32.1 | 51.3 |
| M12S08 | 500752 | 6165445 | 1975 | 149.0 | 67.0 | 120.9 | 74.8 | 31.7 | 2 | 43.4 | 62.2 | 92.6 | 51.6 | 37.0 | 59.4 |
| B12S06 | 501017 | 6165502 | 1975 | 148.5 | 77.1 | 136.4 | 76.6 | 40.3 | 2 | 52.3 | 63.8 | 118.3 | 58.9 | 41.8 | 60.3 |
| B12S15 | 501014 | 6166776 | 1975 | 148.5 | 67.4 | 121.1 | 76.5 | 32.2 | 2 | 44.6 | 68.5 | 97.8 | 52.1 | 39.5 | 62.6 |
| M11S05 | 505662 | 6158933 | 1975 | 149.0 | 62.3 | 113.1 | 72.3 | 30.2 | 1 | 42.4 | 59.1 | 87.9 | 47.0 | 36.0 | 55.1 |
| B12S13 | 501479 | 6167247 | 1975 | 150.8 | 68.2 | 126.0 | 69.4 | 30.5 | 2 | 43.4 | 59.5 | 93.9 | 47.1 | 33.7 | 52.8 |
| M12S01 | 500805 | 6167923 | 1975 | 150.0 | 66.4 | 119.8 | 76.1 | 32.8 | 2 | 42.8 | 67.9 | 96.0 | 51.2 | 38.8 | 61.7 |
| M12S06 | 500824 | 6165905 | 1975 | 150.0 | 74.8 | 130.9 | 76.8 | 34.8 | 2 | 47.3 | 70.6 | 106.8 | 56.9 | 40.2 | 63.1 |
| M25S08 | 652281 | 6205623 | 1975 | 169.0 | 75.5 | 135.5 | 79.2 | 27.0 | 1 | 35.7 | 55.5 | 95.2 | 51.9 | 38.0 | 57.3 |
| M12S05 | 501241 | 6167079 | 1975 | 149.0 | 69.5 | 125.1 | 73.2 | 33.9 | 2 | 45.2 | 65.5 | 102.5 | 52.0 | 37.6 | 59.9 |
| M12S10 | 500820 | 6164336 | 1975 | 149.0 | 65.6 | 115.3 | 72.0 | 30.0 | 2 | 39.5 | 63.3 | 87.5 | 46.8 | 35.9 | 55.8 |
| B12S05 | 501500 | 6165518 | 1975 | 149.0 | 68.0 | 121.5 | 69.2 | 36.3 | 2 | 45.7 | 60.1 | 105.2 | 48.7 | 35.6 | 56.0 |
| B12S20 | 500500 | 6167749 | 1975 | 150.0 | 63.5 | 119.0 | 69.6 | 31.8 | 2 | 42.4 | 56.9 | 99.7 | 46.5 | 34.5 | 53.3 |
| B12S14 | 501485 | 6166757 | 1975 | 149.0 | 72.5 | 128.8 | 76.6 | 34.7 | 2 | 46.4 | 61.1 | 112.1 | 55.7 | 39.8 | 55.6 |
| B12S08 | 500012 | 6165489 | 1975 | 150.0 | 64.4 | 119.3 | 67.0 | 29.1 | 2 | 39.8 | 51.8 | 89.2 | 43.9 | 32.0 | 46.2 |
| M12S02 | 500893 | 6167525 | 1975 | 149.0 | 77.5 | 136.6 | 75.6 | 35.6 | 2 | 47.3 | 63.6 | 108.2 | 59.6 | 41.6 | 58.4 |
| M12S09 | 500906 | 6165184 | 1975 | 149.0 | 57.6 | 106.8 | 65.7 | 29.8 | 2 | 38.9 | 54.0 | 92.2 | 40.5 | 30.4 | 49.1 |
| B12S17 | 499974 | 6166754 | 1975 | 149.3 | 74.4 | 131.6 | 75.3 | 35.0 | 2 | 44.5 | 61.8 | 103.0 | 55.3 | 39.0 | 55.8 |
| B12S10 | 500469 | 6166006 | 1975 | 149.0 | 61.1 | 114.9 | 69.9 | 30.5 | 2 | 40.5 | 57.2 | 97.3 | 45.6 | 32.6 | 51.5 |
| B12S02 | 501534 | 6165985 | 1975 | 150.0 | 61.1 | 113.2 | 65.9 | 32.3 | 2 | 42.4 | 54.1 | 99.8 | 42.4 | 32.1 | 51.7 |
| B12S19 | 499988 | 6167753 | 1975 | 150.4 | 79.5 | 136.3 | 78.2 | 34.4 | 2 | 45.2 | 69.1 | 104.7 | 60.3 | 42.9 | 62.1 |
| B12S16 | 500479 | 6166739 | 1975 | 151.0 | 62.1 | 114.1 | 69.1 | 28.7 | 2 | 38.4 | 57.9 | 90.5 | 43.7 | 33.6 | 54.0 |
| B12S18 | 500002 | 6167290 | 1975 | 152.1 | 80.4 | 135.4 | 75.3 | 36.8 | 2 | 50.2 | 60.0 | 119.5 | 58.2 | 41.6 | 56.0 |
| B12S11 | 501030 | 6167751 | 1975 | 151.0 | 60.7 | 114.4 | 68.5 | 30.0 | 2 | 39.1 | 57.2 | 94.6 | 43.3 | 33.8 | 54.4 |
| B03S04 | 590033 | 6155013 | 1975 | 151.0 | 64.9 | 113.9 | 73.9 | 30.8 | 1 | 39.8 | 56.6 | 98.0 | 47.9 | 37.9 | 56.6 |
| B05S13 | 569993 | 6173498 | 1975 | 154.0 | 57.8 | 114.6 | 70.2 | 25.3 | 2 | 34.8 | 53.3 | 92.6 | 42.8 | 32.8 | 51.0 |
| M12S07 | 500899 | 6165612 | 1975 | 150.0 | 73.5 | 129.4 | 71.9 | 32.0 | 2 | 42.0 | 57.0 | 104.6 | 52.2 | 36.1 | 54.8 |
| B12S03 | 501991 | 6165981 | 1975 | 150.0 | 66.2 | 119.4 | 67.6 | 35.2 | 2 | 42.5 | 54.8 | 111.1 | 45.0 | 34.0 | 50.5 |
| M12S04 | 499959 | 6167022 | 1975 | 150.0 | 68.2 | 121.7 | 69.8 | 33.0 | 2 | 40.8 | 58.9 | 105.1 | 51.7 | 36.7 | 54.7 |
| B12S12 | 501522 | 6167736 | 1975 | 152.0 | 67.3 | 121.2 | 69.8 | 30.8 | 2 | 39.5 | 68.9 | 102.6 | 48.4 | 36.0 | 60.2 |
| M25S09 | 652823 | 6205479 | 1975 | 170.0 | 77.7 | 143.9 | 80.2 | 30.4 | 1 | 37.2 | 61.3 | 107.9 | 57.0 | 39.0 | 61.5 |
| B12S09 | 500026 | 6165987 | 1975 | 150.0 | 69.7 | 123.0 | 67.7 | 34.6 | 2 | 40.6 | 55.8 | 107.7 | 47.9 | 35.0 | 52.7 |
| B05S15 | 569501 | 6173997 | 1975 | 156.6 | 76.5 | 136.9 | 75.3 | 32.7 | 2 | 43.0 | 60.2 | 111.6 | 58.0 | 39.8 | 58.4 |
| B05S14 | 569990 | 6173984 | 1975 | 155.0 | 65.3 | 123.0 | 72.0 | 28.2 | 2 | 36.1 | 54.7 | 99.7 | 48.7 | 35.0 | 53.8 |
| B26S14 | 647373 | 6211396 | 1975 | 160.0 | 61.5 | 113.3 | 68.7 | 27.5 | 2 | 35.7 | 57.2 | 97.6 | 44.6 | 32.7 | 57.9 |
| M28S01 | 660180 | 6282466 | 1975 | 161.0 | 96.5 | 156.1 | 78.9 | 40.8 | 1 | 45.8 | 74.7 | 139.6 | 70.7 | 45.6 | 73.1 |
| B12S01 | 501007 | 6166012 | 1975 | 151.0 | 69.5 | 125.6 | 70.0 | 34.5 | 2 | 44.1 | 58.1 | 123.8 | 49.9 | 35.8 | 52.5 |
| B26S04 | 647228 | 6209993 | 1975 | 163.1 | 63.4 | 116.7 | 68.5 | 28.3 | 2 | 36.1 | 63.2 | 100.8 | 45.5 | 32.8 | 63.4 |
| M03S01 | 591048 | 6155660 | 1975 | 155.0 | 77.5 | 136.1 | 82.2 | 34.8 | 1 | 43.3 | 63.9 | 113.9 | 60.6 | 43.6 | 63.6 |
| M26S01 | 647265 | 6211577 | 1975 | 160.9 | 74.1 | 127.5 | 71.9 | 27.8 | 2 | 35.9 | 66.4 | 99.5 | 52.6 | 36.8 | 68.7 |
| M05S09 | 568383 | 6172576 | 1975 | 157.6 | 74.0 | 135.1 | 74.0 | 29.4 | 2 | 36.6 | 53.7 | 112.8 | 54.8 | 37.7 | 52.0 |
| B21S09 | 504013 | 6337004 | 1975 | 154.0 | 74.6 | 127.3 | 72.6 | 31.0 | 1 | 39.0 | 81.2 | 100.3 | 55.6 | 36.3 | 75.2 |
| B21S01 | 504467 | 6337486 | 1975 | 153.0 | 66.3 | 119.5 | 73.2 | 32.0 | 1 | 43.3 | 73.6 | 102.0 | 52.5 | 36.2 | 72.0 |
| B22S14 | 503514 | 6328487 | 1975 | 156.0 | 74.0 | 128.0 | 72.0 | 31.2 | 1 | 42.1 | 75.9 | 109.1 | 53.0 | 35.1 | 74.1 |
| B21S07 | 503577 | 6337513 | 1975 | 156.0 | 73.5 | 126.3 | 71.5 | 31.0 | 1 | 43.6 | 72.5 | 105.4 | 51.5 | 37.0 | 72.2 |
| B21S02 | 504522 | 6338001 | 1975 | 154.0 | 71.7 | 123.8 | 73.1 | 33.1 | 1 | 43.3 | 75.1 | 104.4 | 54.9 | 37.9 | 74.7 |
| B21S08 | 503469 | 6336997 | 1975 | 154.2 | 73.1 | 127.0 | 71.1 | 31.4 | 1 | 42.5 | 71.5 | 102.6 | 53.0 | 36.9 | 73.4 |
| B22S17 | 503478 | 6327016 | 1975 | 155.9 | 70.1 | 122.0 | 69.9 | 32.2 | 1 | 43.1 | 73.2 | 103.5 | 51.2 | 35.8 | 70.4 |
| M21S04 | 503612 | 6337366 | 1975 | 155.0 | 71.0 | 122.4 | 72.1 | 31.1 | 1 | 39.2 | 71.4 | 104.9 | 50.4 | 34.4 | 70.0 |
| M22S07 | 506588 | 6328220 | 1975 | 155.0 | 81.9 | 137.9 | 73.0 | 33.0 | 1 | 43.0 | 81.5 | 112.6 | 59.9 | 38.3 | 81.0 |
| M22S03 | 503159 | 6328153 | 1975 | 154.0 | 74.2 | 127.4 | 71.6 | 33.7 | 1 | 42.9 | 72.8 | 108.1 | 52.9 | 35.5 | 72.4 |
| B22S13 | 503483 | 6328988 | 1975 | 156.0 | 70.4 | 124.7 | 68.6 | 33.4 | 1 | 42.2 | 72.2 | 114.1 | 48.8 | 35.0 | 70.7 |
| B22S15 | 503527 | 6327987 | 1975 | 155.0 | 77.9 | 131.0 | 71.6 | 35.5 | 1 | 43.6 | 79.6 | 120.0 | 53.4 | 37.0 | 78.5 |
| M22S06 | 506012 | 6327833 | 1975 | 156.0 | 80.8 | 137.3 | 73.0 | 33.5 | 1 | 43.6 | 80.1 | 120.7 | 58.2 | 38.8 | 80.9 |
| B22S18 | 503967 | 6327015 | 1975 | 155.0 | 71.4 | 127.9 | 72.3 | 34.6 | 1 | 44.2 | 74.2 | 115.4 | 56.9 | 38.2 | 75.0 |
| B22S16 | 503482 | 6327554 | 1975 | 154.0 | 78.2 | 133.0 | 71.6 | 34.3 | 1 | 46.0 | 77.4 | 116.6 | 57.0 | 38.1 | 75.2 |
| B22S11 | 504018 | 6328495 | 1975 | 155.0 | 80.0 | 137.0 | 72.2 | 33.5 | 1 | 43.1 | 74.3 | 115.6 | 56.7 | 38.1 | 75.6 |
| M22S08 | 505799 | 6329418 | 1975 | 156.0 | 77.4 | 133.9 | 69.2 | 33.6 | 1 | 42.7 | 72.6 | 120.3 | 55.1 | 36.4 | 74.3 |
| M22S02 | 504305 | 6326402 | 1975 | 156.0 | 78.6 | 132.8 | 72.2 | 32.0 | 1 | 43.1 | 70.7 | 112.2 | 55.2 | 37.9 | 71.5 |
| B22S03 | 505495 | 6329000 | 1975 | 156.0 | 79.9 | 131.8 | 70.8 | 33.8 | 1 | 44.1 | 73.3 | 114.5 | 57.4 | 37.5 | 73.3 |
| B22S01 | 505502 | 6328052 | 1975 | 157.0 | 80.2 | 132.4 | 70.1 | 32.3 | 1 | 40.4 | 68.0 | 108.9 | 54.4 | 36.9 | 69.7 |
| M21S06 | 504548 | 6337216 | 1975 | 154.1 | 70.4 | 121.6 | 70.4 | 32.5 | 1 | 41.9 | 66.1 | 104.6 | 49.5 | 35.5 | 68.1 |
| B22S12 | 504000 | 6329018 | 1975 | 156.0 | 77.9 | 132.3 | 69.8 | 32.0 | 1 | 44.0 | 66.4 | 113.1 | 52.9 | 36.0 | 70.0 |
| B22S05 | 505003 | 6328499 | 1975 | 155.3 | 82.4 | 136.3 | 70.6 | 34.5 | 1 | 43.8 | 70.6 | 118.0 | 57.6 | 36.8 | 72.0 |
| M22S01 | 504999 | 6326379 | 1975 | 155.4 | 81.6 | 141.3 | 73.0 | 35.4 | 1 | 44.1 | 73.1 | 123.9 | 57.8 | 37.5 | 72.9 |
| B22S02 | 505506 | 6328507 | 1975 | 156.0 | 76.2 | 131.7 | 72.2 | 34.2 | 1 | 44.5 | 69.6 | 121.4 | 57.5 | 38.9 | 72.4 |
| M22S04 | 504333 | 6328116 | 1975 | 157.1 | 76.4 | 132.0 | 72.2 | 34.7 | 1 | 43.1 | 69.2 | 118.7 | 53.0 | 36.8 | 69.4 |
| B22S07 | 505000 | 6327500 | 1975 | 155.0 | 80.3 | 134.5 | 70.5 | 32.5 | 1 | 42.0 | 67.0 | 111.8 | 55.5 | 37.5 | 68.3 |
| M22S05 | 505237 | 6327935 | 1975 | 156.0 | 80.4 | 134.2 | 68.3 | 32.9 | 1 | 42.0 | 66.4 | 110.5 | 53.9 | 35.0 | 66.5 |
| B22S20 | 503972 | 6328007 | 1975 | 156.1 | 73.3 | 128.0 | 70.2 | 32.7 | 1 | 42.6 | 65.0 | 112.9 | 51.5 | 36.2 | 67.5 |
| M22S09 | 504861 | 6329544 | 1975 | 154.0 | 75.1 | 123.9 | 68.3 | 29.8 | 1 | 38.4 | 62.9 | 101.9 | 53.2 | 35.8 | 62.4 |
| B22S10 | 505495 | 6327508 | 1975 | 156.6 | 83.1 | 139.0 | 71.7 | 33.8 | 1 | 41.8 | 70.9 | 126.2 | 60.1 | 38.0 | 69.6 |
| B21S10 | 504523 | 6336902 | 1975 | 154.8 | 76.6 | 130.9 | 71.2 | 31.6 | 1 | 40.9 | 64.0 | 103.9 | 53.8 | 36.4 | 65.7 |
| B22S09 | 505500 | 6326940 | 1975 | 156.6 | 75.1 | 131.3 | 73.5 | 32.5 | 1 | 42.0 | 65.9 | 115.2 | 55.2 | 37.0 | 66.9 |
| M22S10 | 504006 | 6329614 | 1975 | 157.0 | 82.1 | 138.6 | 70.3 | 33.2 | 1 | 41.3 | 66.6 | 119.7 | 57.6 | 37.2 | 67.2 |
| M21S05 | 504004 | 6337322 | 1975 | 155.0 | 73.5 | 126.4 | 72.2 | 31.6 | 1 | 40.4 | 63.7 | 103.8 | 55.5 | 37.2 | 62.8 |
| B22S19 | 504004 | 6327518 | 1975 | 155.5 | 80.5 | 134.5 | 72.7 | 32.9 | 1 | 45.4 | 65.9 | 115.1 | 55.3 | 36.4 | 64.4 |
| B22S08 | 505014 | 6327007 | 1975 | 156.0 | 80.9 | 137.8 | 71.2 | 32.2 | 1 | 42.5 | 64.5 | 115.0 | 54.9 | 36.4 | 64.0 |
| B22S06 | 504987 | 6327943 | 1975 | 155.2 | 83.5 | 144.0 | 71.3 | 34.0 | 1 | 43.7 | 64.6 | 116.0 | 59.4 | 38.5 | 67.3 |
| B22S04 | 505003 | 6328993 | 1975 | 155.1 | 84.9 | 143.3 | 71.8 | 33.7 | 1 | 43.1 | 66.5 | 119.7 | 60.6 | 38.4 | 65.8 |
| B07S20 | 561749 | 6157013 | 1977 | 156.0 | 65.4 | 119.3 | 73.9 | 27.5 | 1 | 36.0 | 49.0 | 96.4 | 45.9 | 36.0 | 49.9 |
| B07S01 | 560244 | 6158003 | 1977 | 155.0 | 66.1 | 122.2 | 72.3 | 26.8 | 2 | 35.0 | 52.9 | 99.1 | 45.4 | 34.0 | 50.2 |
| M07S01 | 559802 | 6157270 | 1977 | 154.6 | 63.8 | 114.6 | 73.1 | 29.7 | 1 | 36.8 | 56.4 | 95.4 | 46.1 | 36.3 | 54.4 |
| M07S09 | 561479 | 6155808 | 1977 | 154.7 | 61.3 | 109.6 | 70.7 | 28.5 | 1 | 36.6 | 66.7 | 88.1 | 43.0 | 33.7 | 65.2 |
| M07S10 | 561219 | 6155762 | 1977 | 154.0 | 59.0 | 109.1 | 69.0 | 26.3 | 1 | 35.7 | 63.1 | 90.4 | 41.0 | 32.0 | 62.5 |
| B07S11 | 561271 | 6156028 | 1977 | 155.0 | 70.6 | 128.3 | 77.4 | 32.5 | 1 | 40.1 | 71.5 | 105.0 | 54.2 | 40.1 | 70.4 |
| B07S16 | 560227 | 6157506 | 1977 | 155.0 | 60.2 | 109.4 | 71.4 | 27.2 | 1 | 36.3 | 60.7 | 89.1 | 43.2 | 33.9 | 63.2 |
| B07S12 | 560769 | 6155989 | 1977 | 154.0 | 62.0 | 115.9 | 75.6 | 30.6 | 1 | 39.0 | 63.0 | 94.5 | 47.8 | 35.9 | 62.3 |
| B07S14 | 560233 | 6156516 | 1977 | 155.8 | 68.7 | 121.8 | 75.8 | 31.0 | 1 | 40.8 | 64.7 | 101.3 | 51.2 | 37.0 | 64.5 |
| B07S02 | 559591 | 6157926 | 1977 | 155.0 | 64.4 | 118.0 | 75.3 | 31.0 | 1 | 41.2 | 52.8 | 98.6 | 47.3 | 36.1 | 55.4 |
| B07S13 | 560248 | 6155978 | 1977 | 156.6 | 63.6 | 117.2 | 69.6 | 27.5 | 2 | 34.4 | 53.8 | 95.4 | 45.0 | 32.2 | 55.3 |
| M07S07 | 562444 | 6157619 | 1977 | 155.0 | 78.1 | 133.7 | 77.0 | 32.8 | 1 | 43.4 | 56.7 | 107.3 | 57.4 | 41.4 | 56.8 |
